# Infrared Imaging for Autonomous Power Inspection: A Review from Detector to System Integration

**DOI:** 10.3390/s26113552

**Published:** 2026-06-03

**Authors:** Yingye Guo, Yuxi Du, Run Mao, Yongyin Zhao, Junxiong Guo

**Affiliations:** 1 College of Overseas Education, Chengdu University, Chengdu 610106, China; guoyingye@cdu.edu.cn (Y.G.); duyuxi@cdu.edu.cn (Y.D.); 2 School of Electronic Information and Electrical Engineering, Chengdu University, Chengdu 100106, China; maorun@cdu.edu.cn; 3 Institute for Advanced Study, Chengdu University, Chengdu 100106, China

**Keywords:** infrared imaging, automatic inspection, power systems, infrared detectors, signal processing, unmanned aerial vehicles (UAVs), robotics, internet of things (IoT)

## Abstract

The transition toward smart grids and Industry 4.0 demands a fundamental shift in maintenance strategies, as manual inspection methods are increasingly being supplanted by automated monitoring systems. Among the advanced technologies for smart inspection, infrared imaging has advantages including non-contact operation, intuitive visualization, and predictive capabilities, which has become a cornerstone for autonomous inspection of critical power infrastructure. This review provides recent advancements in infrared imaging, with a specific focus on automated power system inspection. The discussion starts with an overview of the fundamental principles and system architectures, emphasizing the pivotal role of infrared detectors. A detailed analysis traces the technological evolution from traditional photon detectors to current uncooled microbolometers, and critically assesses emerging low-dimensional materials. The analysis highlights inherent performance trade-offs among sensitivity, operating temperature, and fabrication cost. Subsequently, the review explores advanced signal processing algorithms, such as real-time non-uniformity correction and adaptive noise suppression, which are typically implemented on FPGA platforms. Advanced optical configurations—encompassing computational imaging, lensless designs, and scattering suppression methods—are also discussed, demonstrating how their convergence enhances image fidelity and operational reliability in complex field environments. Representative application paradigms are surveyed, including drone-based transmission line inspections, patrol robots in substations, and fault diagnosis in photovoltaic plants; for each, operational efficacy and economic benefits are assessed. Despite considerable progress, several challenges persist, notably the performance–stability–cost trilemma in novel detector development, the substantial computational demands of end-to-end optimized systems, and a lack of standardization. Finally, the review outlines future research directions, such as high-performance uncooled arrays, AI-driven co-design of optics and algorithms, and the development of standardized, low-cost, intelligent inspection platforms.

## 1. Introduction

The reliable and efficient operation of power grids is fundamental to economic stability and public safety. As the backbone of modern industrial society, power systems rely on continuous monitoring of critical assets, including transmission lines, substations, and photovoltaic plants [1,2]. Conventional maintenance strategies predominantly depend on manual inspections and periodic scheduling, which are labor-intensive, time-consuming, and constrained by human subjectivity and limited accessibility. More importantly, such approaches lack continuous monitoring capability, making it difficult to detect early-stage faults, such as overheating connections, insulation degradation, and incipient component failures. These undetected anomalies may accumulate and ultimately lead to severe outages, causing substantial economic losses and safety risks [3].

With the rapid development of the Industrial Internet of Things (IIoT) and Industry 4.0, power system maintenance is transitioning toward autonomous, data-driven, and predictive paradigms [4,5,6,7,8]. Automatic inspection systems deployed on platforms such as unmanned aerial vehicles (UAVs) [9,10,11] and ground robots [12] have therefore emerged as an effective solution. Such automatic systems enable continuous operation, higher inspection frequency, and access to hazardous or remote environments, they therefore significantly improve inspection efficiency and reliability. However, their performance critically depends on the sensing modality used for fault detection. Among various non-destructive testing techniques, infrared (IR) imaging has become a key enabling technology due to its non-contact operation, intuitive visualization, and capability for early anomaly detection. By capturing the thermal radiation emitted from object surfaces, IR imaging provides quantitative temperature distribution information, which allows inspectors to identify abnormal thermal patterns that are strongly correlated with electrical faults. Compared with conventional inspection methods, IR imaging offers superior adaptability to complex environments and supports real-time condition monitoring.

To date, infrared thermography has been widely applied in power systems; however, its integration into fully autonomous inspection frameworks remains an active and rapidly evolving research interest [13], an evolution that is driven by the convergence of advances in infrared detector technology, computational imaging and signal processing, and intelligent system integration. While infrared detectors have progressed from bulky, cryogenically cooled photon detectors to compact uncooled microbolometers, emerging low-dimensional material-based sensors present new opportunities for performance enhancement. Meanwhile, real-time signal processing algorithms implemented on platforms such as field-programmable gate arrays (FPGAs) enable effective correction of non-uniformity, suppression of noise, and extraction of reliable diagnostic information from raw data [14,15,16]. In parallel, the development of advanced optical configurations, including scattering suppression and lensless imaging, together with the co-design of optics and perception algorithms, has significantly improved system robustness and adaptability in mobile inspection scenarios [17,18].

Here, we review the technical foundation of infrared imaging technology, focusing on its core role in automatic power inspection. We explore its typical application scenarios and predict future technological breakthroughs and challenges. The structure of this review is as follows: Section 2 describes the basic physical principles of infrared imaging technology and covers the overall architecture of the autonomous inspection system. Section 3 provides an analysis of infrared detector technology—the core of perception—covering its evolution, performance comparison, and emerging material applications. Section 4 discusses advanced signal processing and computational imaging methods that improve image quality and diagnostic reliability. Section 4 also introduces the design concept of optical systems and explains their preprocessing capability in complex environments. Section 5 analyzes system integration schemes and examines the application efficiency of UAVs and robots in typical scenarios, including transmission lines, substations, and photovoltaic (PV) power stations. Section 6 discusses major challenges at the material, algorithm, system integration, and industrialization levels, and also looks forward to future trends. Finally, Section 7 summarizes the key views of the full text for the construction of intelligent power operation and maintenance systems.

## 2. Technical Basis of Infrared Imaging Autonomous Power Patrol Inspection

**Figure 1:**
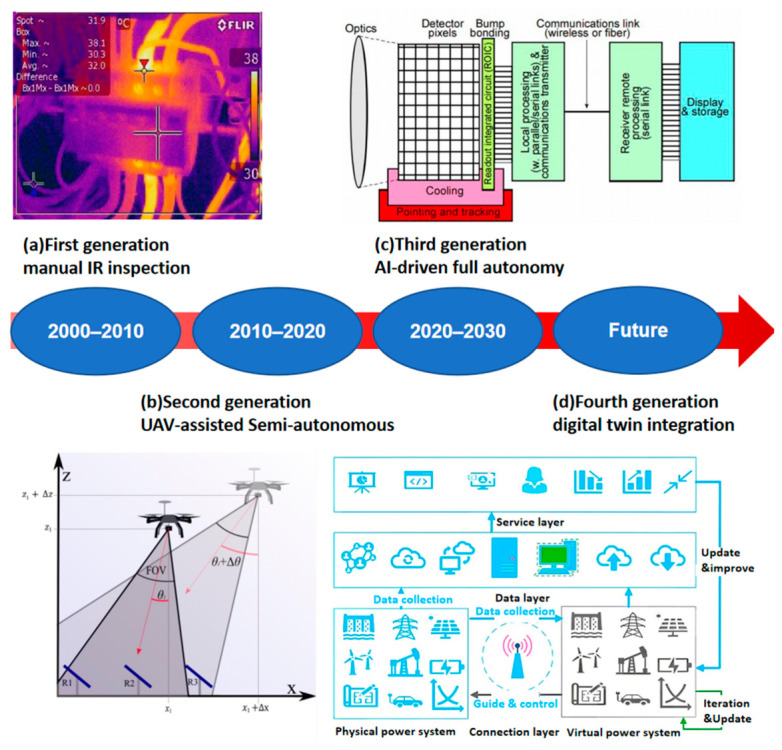
Schematic diagram of the evolution of power inspection technology, showing the four generations of IR inspection methods, along with representative technical architectures and applications. (**a**) Thermal imaging hot spot map of a distribution board (Ref. [19]). (**b**) Schematic diagram of UAV camera field of view (FOV) variation with flight altitude and attitude, illustrating the geometric relationship between imaging parameters and target regions (Ref. [20]). (**c**) Schematic diagram of an imaging system showing key subsystems (Ref. [21]). (**d**) Simplified architecture of digital twin (DT) technology in power systems (Ref. [22]).

Infrared imaging-based autonomous power inspection integrates infrared sensing, intelligent data processing, and robotic platforms to achieve non-contact, real-time fault diagnosis and condition monitoring of electrical equipment. Its technical foundation relies on both the physical principles of infrared radiation and the system-level architecture that enables autonomous operation. From a developmental perspective, infrared inspection technology has evolved from manual inspection to fully autonomous systems. As illustrated in Figure 1, current solutions represent an AI-driven stage characterized by intelligent perception, real-time processing, and platform integration, with UAV-based inspection systems being among the most representative implementations.

### 2.1. Working Principle of Infrared Imaging

Since all electrical equipment with a temperature above absolute zero (T > 0 K) continuously emits infrared radiation [23], Planck’s law describes the relationship between radiation power, wavelength, and temperature, which is formulated as $M(\lambda ,T)=\frac{C_{1}}{\lambda^{5}\left(e^{\frac{C_{2}}{\lambda T}}-1\right)}$, where M(λ,T) is the spectral radiant exitance, T is the device temperature (K), λ is the radiation wavelength (μm), C1 and C2 are constants [19,23]. For the common operating temperature range of electrical equipment (−40–1000 °C), the peak wavelength of its infrared radiation follows Wien’s displacement law${\;\lambda }_{\mathrm{peak}}\;=\;\frac{0.0029}{T}$, according to which the peak radiation wavelength falls mainly in the mid-wave infrared (MWIR, 3–5 μm) and long-wave infrared (LWIR, 8–14 μm) bands [19,21]. Although infrared radiation encounters significant absorption and scattering during atmospheric transmission, the MWIR and LWIR bands act as atmospheric windows with transmittance typically ranging from 70% to 90%, making them the primary operational bands for power inspection systems [19,23]. In contrast, strong absorption in the 5–7.5 μm range significantly limits signal transmission. Compared with visible light, infrared radiation is less susceptible to scattering by small particles, allowing operation under low-visibility conditions such as fog or nighttime. However, under severe weather conditions, including heavy rain or dense fog, transmission losses become non-negligible and must be mitigated through system-level parameter optimization [21,24,25].

Beyond the mid-wave and long-wave infrared bands discussed above, the short-wave infrared (SWIR) band (typically 0.9–1.7 μm, extending to 400 nm when combined with visible light into a broad spectrum) increasingly contributes to power inspection and related industrial fields. The unique physical characteristics of the SWIR band—namely strong penetration through silicon and high water absorption—complement the detection capabilities of MWIR and LWIR bands. As with other infrared bands, objects above absolute zero emit SWIR radiation, whose spectral distribution is likewise governed by Planck’s law. For electrical equipment and industrial components, SWIR radiation reveals surface defects, material inhomogeneities, and subtle temperature variations often undetectable by MWIR or LWIR imaging. A corresponding atmospheric window, which maintains relatively high transmittance (usually 60–85%) under normal conditions, enables highly effective long-distance detection. Notably, broadband short-wave infrared imaging (covering 400–1700 nm, integrating visible light and SWIR) further expands the detection range, capturing both visible surface features and infrared thermal information and thus bridging the gap between traditional infrared and visible light inspection.

Infrared detectors serve as the core components that convert incident radiation into electrical signals [5,26,27]. Based on their operating mechanisms, they are generally classified into photodetectors and thermal detectors. Photodetectors, such as HgCdTe and InSb, achieve high sensitivity and fast response by exploiting photon–electron interactions, but require cryogenic cooling, resulting in increased system complexity and cost [19,21,23]. In comparison, thermal detectors, including microbolometers and pyroelectric devices, operate at or near room temperature by converting absorbed radiation into heat-induced physical changes. Although their sensitivity is relatively lower, their compact size, low cost, and ease of integration make them the dominant choice in practical autonomous inspection systems [19,23]. Recent advances in microbolometer arrays have enabled higher resolution and improved imaging performance, supporting detailed visualization of equipment conditions.

The generation of infrared images involves multiple processing steps, including signal amplification, analog-to-digital conversion, and pseudo-color mapping [19,21,28]. Temperature calibration establishes the relationship between grayscale values and actual temperature using reference sources, ensuring measurement accuracy. Additional processing techniques, such as image fusion with visible light data and automated hot spot detection, further enhance fault localization and diagnostic reliability [19,29].

### 2.2. Infrared Imaging Autonomous Power Inspection System Architecture

An autonomous infrared inspection system typically consists of integrated sensing, control, processing, communication, and power subsystems. First, the sensing module, which integrates custom infrared optics, focal plane arrays (FPAs), and high-speed preprocessing circuits, captures thermal radiation and converts it into temperature images. Designers select specialized infrared lens materials, such as germanium, zinc selenide, and zinc sulfide, based on their specific spectral transmission properties in MWIR or LWIR bands [25]. Detector arrays, typically based on FPA architectures, provide spatially resolved temperature information, while preprocessing circuits enhance signal quality through amplification and noise reduction.

Second, the autonomous motion control subsystem ensures accurate navigation and stable imaging during inspection. By integrating positioning technologies such as GPS/BeiDou and inertial measurement units (IMUs), the system achieves high-precision localization. Path planning algorithms generate optimized inspection trajectories based on infrastructure layouts, while stabilization mechanisms, including multi-axis gimbals, compensate for platform motion to maintain image quality. Obstacle avoidance capabilities, enabled by LiDAR and vision sensors, enhance operational safety in complex environments [24,30,31].

Third, the data processing and analysis subsystem plays a central role by running real-time algorithms that execute temperature calibration, image enhancement, hot spot extraction, and fault classification. Machine learning algorithms, such as support vector machines and convolutional neural networks, are employed to identify fault types and assess severity based on thermal patterns and historical data [19,21,31]. These capabilities allow automated decision-making and significantly reduce reliance on manual interpretation.

Fourth, the communication subsystem supports real-time data transmission and remote interaction. Wireless technologies, including 4G/5G and Wi-Fi, enable transmission of infrared images and diagnostic information to control centers, while satellite communication provides coverage in remote areas [24]. Local communication interfaces facilitate on-site data access and system configuration. To ensure reliable operation, the communication system must satisfy stringent requirements for low latency and high transmission reliability.

Fifth, the power and auxiliary subsystem provides energy supply and environmental support for the entire system. Depending on the platform, energy sources may include high-density batteries or hybrid solutions such as solar-assisted power systems. Auxiliary components, including environmental sensors and safety protection mechanisms, provide additional information for temperature calibration and ensure safe operation under varying conditions [19,21,24].

## 3. Infrared Detector Technology

Infrared detectors constitute the core sensing components of autonomous power inspection systems, directly determining the capability to identify thermal anomalies, evaluate fault severity, and ensure reliable operation under complex environments [32]. In practical scenarios such as UAV-based inspection, substation robotics, and long-distance transmission monitoring [33], detectors convert weak thermal radiation emitted by electrical equipment into measurable electrical signals, which underpins the entire perception pipeline from anomaly localization to AI-based fault diagnosis [34]. Consequently, detector performance not only affects sensitivity and response speed but also constrains system-level attributes, including power consumption, payload capacity, and deployment scalability. Because autonomous platforms impose stricter requirements on miniaturization, energy efficiency, and environmental adaptability than conventional inspection systems do, detector technology has become the primary driver of system evolution [35,36,37,38,39].

### 3.1. Evolution of Infrared Detector Technology: From Performance-Driven to System-Oriented Design

**Figure 2:**
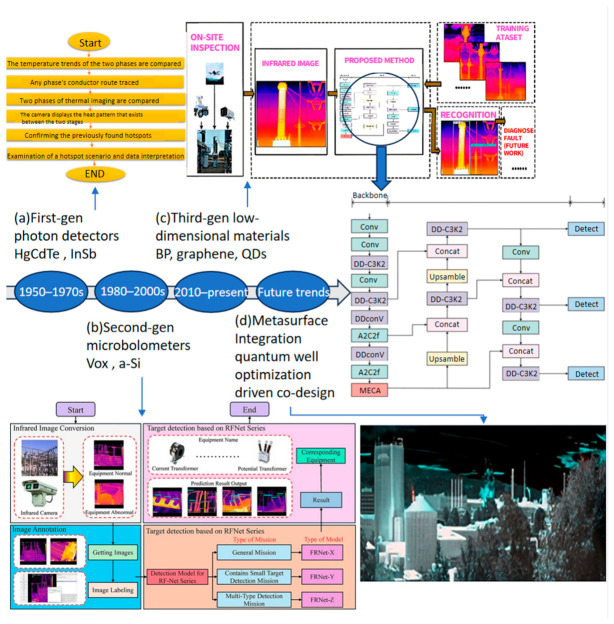
A schematic diagram of the technological development of infrared detectors, showing key generations, representative materials, and future trends of imaging systems. (**a**) Flowchart of infrared hot spot inspection and diagnosis procedure for power equipment, including multi-phase temperature comparison, thermal imaging analysis, and hot spot verification (Ref. [45]). (**b**) RFNet-based intelligent infrared target detection workflow for power substation equipment (Ref. [46]). (**c**) Framework of the proposed intelligent infrared fault diagnosis method for power equipment, covering on-site inspection, dataset construction, deep learning-based recognition, and future fault diagnosis applications (Ref. [47]). (**d**) Bispectral infrared image of an industrial site taken with a 384 × 288 dual-color InAs/GaSb SL camera. The two-color channels 3–4 μm and 4–5 μm are represented by the complementary colors, cyan and red, respectively (Ref. [43]).

The development of infrared detectors has transitioned from a purely performance-driven paradigm toward a system-oriented co-optimization framework (Figure 2). While early efforts focused on maximizing sensitivity through photon-based detection mechanisms, recent advances increasingly emphasize the complex trade-offs among sensitivity, cost, operating conditions, and integration compatibility.

First-generation detectors, represented by cryogenically cooled photon devices such as HgCdTe and InSb, established the benchmark for high-sensitivity infrared detection [40,41,42]. By exploiting bandgap engineering, these materials enable broad spectral coverage and millikelvin-level temperature resolution, allowing them to support high-precision applications such as internal defect detection in transformers and long-distance inspection of transmission infrastructure [41,43,44]. However, their reliance on cryogenic cooling (typically around 77 K) leads to bulky system design, high power consumption, and substantial cost, which severely limits their deployment in mobile and large-scale inspection systems.

The emergence of uncooled microbolometers in the 1990s marked a critical shift toward practical deployment. Based on thermally induced resistance changes in materials such as VOx and amorphous silicon, these detectors operate at room temperature and significantly reduce system complexity [40,41]. Advances in micro-electro-mechanical system (MEMS) fabrication have enabled continuous improvements in pixel size, array resolution, and manufacturing cost. As a result, uncooled detectors have become the dominant solution for autonomous inspection, enabling large-scale deployment on UAVs and robotic platforms. Despite their advantages in cost and portability, low sensitivity and slow response speed relative to photon detectors bring inherent limitations, particularly in long-range detection and weak signal scenarios.

Recent developments have ushered in a third stage characterized by hybrid integration and emerging materials. On the one hand, advanced photon detectors such as quantum well infrared photodetectors (QWIPs) and type II superlattices improve material uniformity, reduce cooling requirements, and enhance reliability [48]. On the other hand, integrating computational imaging and AI-based processing compensates for hardware limitations, effectively extending the performance envelope of uncooled detectors [49]. Simultaneously, low-dimensional materials—including two-dimensional (2D) materials and quantum dots—have emerged as promising candidates for next-generation detectors, offering opportunities to break conventional trade-offs between sensitivity, cost, and operating temperature [40,41,50].

### 3.2. Emerging Low-Dimensional Material-Based Detector

Low-dimensional materials introduce fundamentally new mechanisms for infrared detection by leveraging quantum confinement, tunable band structures, and enhanced light–matter interactions. Such unique properties enable flexible spectral response, high carrier mobility, and compatibility with novel device architectures, making low-dimensional materials key enablers for next-generation intelligent inspection systems.

#### 3.2.1. Two-Dimensional Materials

Two-dimensional materials exhibit unique advantages because of their atomically thin structures and strong electro-optical tunability [51,52,53,54]. For instance, black phosphorus provides a thickness-dependent bandgap and intrinsic anisotropy, enabling broadband infrared response and dynamic spectral tuning. Its capability to operate near room temperature while maintaining relatively high sensitivity makes it suitable for lightweight, high-precision inspection platforms [40,41,49]. Graphene, with its ultra-high carrier mobility and broadband absorption, offers exceptional response speed and flexibility, supporting applications that require the detection of transient or weak thermal signals [40,41,50]. When combined with AI algorithms, graphene-based detectors can perform multi-band cooperative detection, improving fault identification accuracy in complex environments [55,56,57,58]. However, low intrinsic absorption necessitates the integration with nanophotonic structures to enhance efficiency. Transition metal dichalcogenides [59,60], such as MoS_2_ and WSe_2_, enable high-performance heterojunction devices with improved carrier separation and low-power operation, which are particularly attractive for distributed and long-duration monitoring scenarios [55,56,57,58,59]. Stacking two-dimensional materials with different bandgaps forms van der Waals heterojunctions [61]. Heterojunction engineering widens the spectral response range and efficiently separates photogenerated carriers, thereby enhancing detector sensitivity and response speed. These detectors can operate in self-powered mode at room temperature, which significantly reduces power consumption of autonomous inspection equipment and extends operational endurance. Such superior performance makes them particularly suitable for long-term autonomous monitoring of transmission lines in remote areas. Furthermore, detection performance can be further optimized through local doping [62] or defect engineering [63] and asymmetric contact engineering [64], all of which provide considerable optimization potential. For example, introducing defect energy levels in MoS_2_ via N_2_ plasma doping enables near-infrared light detection [65]. Alternatively, using metals with different work functions as source and drain electrodes forms asymmetric Schottky barriers [66], achieving self-powered detection and expanded spectral range [26].

#### 3.2.2. Quantum Dot Materials: A New Path for Low-Cost Wide-Spectrum Detection

Quantum dot materials [40,41,50] (e.g., PbS and PbSe) leverage the quantum size effect, which allows their bandgaps to be precisely tailored by controlling particle size, enabling broad spectral response from the near-infrared to the mid-infrared range. These materials are simple and low-cost to produce, and can be coated on flexible substrates over large areas using solution-based methods, thus being suitable for mass production. The core advantage of quantum dot infrared detectors lies in the synergy between spectral tunability and low cost. They possess a high optical absorption coefficient, approximately 10^5^ cm^−1^, which enables effective capture of weak thermal radiation signals from power equipment. The device structure remains simple and easily integrates with silicon-based CMOS circuits, enabling monolithic integration of the detector and signal processing units and thus reducing overall system volume. In autonomous power inspection scenarios, quantum dot detectors can serve as a low-cost alternative to uncooled microbolometers and are suitable for use in distributed power monitoring nodes. Heterogeneous integration with two-dimensional materials (e.g., MoS_2_/PbS QDs) offers further improvements, as heterojunction engineering optimizes band alignment to enhance sensitivity and response speed, which enables high-precision thermal imaging of equipment such as cable joints and switchgear. The flexible nature of quantum dot materials allows fabrication of curved detectors, which are suitable for integration with irregular carriers like drones and inspection robots, expanding the coverage of autonomous inspections. Currently, the stability and long-term operational reliability of quantum dot detectors still require improvement. However, their low cost and broad spectral response make them a feasible option for large-scale deployment of infrared imaging systems in autonomous power inspections.

### 3.3. Trade-Offs Between Different Types of Detectors

The diversity of autonomous power inspection scenarios—including long-range patrols, close-range diagnostics, continuous monitoring, and portable detection—imposes heterogeneous performance requirements on infrared detectors. Sensitivity, operating temperature, and cost are inherently interdependent, forming a fundamental trade-off framework that governs detector selection. Different technological routes exhibit complementary characteristics, enabling scenario-oriented adaptation. Table 1 summarizes the key performance metrics and application suitability of representative detector types, revealing the inherent trade-offs among sensitivity, cost, and operating conditions. The cooled photon detectors (HgCdTe, InSb, QWIP) achieve ultra-high sensitivity (NETD ≤ 0.02 °C) but require high manufacturing costs (40–80 k RMB) and strict cooling requirements. In contrast, uncooled microbolometers and quantum dot detectors feature low cost (≤8 k RMB) and room temperature operation, but deliver relatively lower sensitivity (NETD ≥ 0.03 °C). Black phosphorus detectors, as an emerging type, balance sensitivity (NETD = 0.025 °C) and cost (20 k RMB), demonstrating the potential to break the traditional sensitivity–cost trade-off. These data directly verify the nonlinear trade-off relationship between sensitivity and cost mentioned above, and provide a direct basis for selecting detectors according to application scenarios.

Refrigerated photon detectors, such as HgCdTe (response band 3–5 μm), InSb (response band 1–5 μm), and QWIP-based (response band 3–12 μm) devices (see Table 1), prioritize sensitivity and are therefore indispensable in high-end inspection scenarios requiring long-distance detection and deep fault diagnosis. Their wide response bands (especially QWIP’s 3–12 μm) enable effective capture of weak thermal signals from long distances, making these detectors suitable for internal defect inspection of GIS equipment and high-precision thermal analysis of transformers. In autonomous systems, these detectors are typically integrated into large UAV platforms or ground-based inspection vehicles equipped with dedicated cooling subsystems, enabling kilometer-scale, high-resolution thermal imaging [40,41].

In contrast, uncooled microbolometers have become the dominant solution for large-scale deployment due to room temperature operation, low cost (only 8 k RMB, see Table 1), and compact form factor. Compared with cooled photon detectors (40–80 k RMB in Table 1), uncooled microbolometers reduce relevant cost to one-tenth to one-fifth of that of cooled devices, supporting high compatibility with UAVs, portable devices, and distributed monitoring nodes [48]. Relatively lower sensitivity and slower response speed restrict the capability to capture subtle temperature variations and transient fault signatures. Such inherent constraints confine uncooled microbolometers to routine inspection tasks, such as hot spot detection in transmission lines, substations, and photovoltaic systems. Recent advances in pixel miniaturization and AI-assisted computational imaging have partially mitigated the above limitations by enhancing effective resolution and signal interpretability, thus broadening the application scope of uncooled detectors [40,41].

Emerging detectors based on low-dimensional materials, including two-dimensional materials and quantum dots, offer a promising pathway to break the conventional performance–cost trade-off. Operating at or near room temperature without complex cooling infrastructure, such devices can achieve sensitivity comparable to that of mid-range cooled detectors. These devices can operate at or near room temperature without complex cooling infrastructure, while potentially achieving sensitivity comparable to mid-range cooled detectors. In addition to improved response speed, they provide unique advantages such as spectral tunability and flexible integration, which are particularly attractive for next-generation intelligent inspection systems [40,41,50]. For instance, black phosphorus detectors enable broadband infrared detection with enhanced sensitivity, whereas hybrid quantum dot–2D material heterostructures support multi-band sensing within a single platform. Despite these advantages, challenges related to large-area uniformity, long-term stability, and scalable fabrication remain significant barriers to industrial adoption.

### 3.4. Contributions of Detector Technologies to Inspection Systems

In summary, the generational evolution of infrared detector performance directly defines the application boundaries and operational paradigms of autonomous inspection systems. This contribution from detector technology to system capability can be understood at three levels:

(1) High-sensitivity cooled photon detectors (e.g., HgCdTe, InSb), with their millikelvin-level temperature resolution, provide the physical foundation for deep-level fault diagnosis inside transformer windings and long-range high-precision inspection, making them the core sensing source for high-end precision diagnostic scenarios.
(2) The maturation and cost reduction in uncooled microbolometers have been pivotal in shifting the inspection paradigm from manual to unmanned platforms. Their compact size and low power consumption overcome payload limitations, enabling large-scale, high-frequency routine inspections via UAVs and ground robots, thereby directly facilitating the widespread adoption of inspection paradigms discussed in Section 5.
(3) Emerging detectors based on low-dimensional materials, such as 2D materials and quantum dots, aim to break the inherent trade-off among high performance, low cost, and high stability. Their spectral tunability and flexible integration potential promise a new generation of autonomous inspection systems capable of multi-spectral collaborative diagnosis and mounting on irregularly shaped carriers.

## 4. Integrated Application of Advanced Signal Processing and Optical Systems

### 4.1. Signal Processing Technology

Signal post-processing serves as the technological bridge that transforms detector physical performance (Section 3) into the system-level diagnostic capability required by autonomous inspection platforms (Section 5). By compensating for detector hardware shortcomings in non-uniformity, noise, and dynamic range, it enhances image quality and ensures reliable fault diagnosis in complex field environments. Its core goal is to resolve inherent issues in infrared images, including non-uniformity and random noise, through algorithm optimization. The technology must also accommodate differentiated requirements of cooled detectors, uncooled detectors, and wide-spectrum short-wave infrared detectors. Table 2 compares key performance indicators and applicable scenarios of various signal processing algorithms, quantifying the trade-offs among latency, processing effect, and hardware requirements.

Due to limitations in manufacturing processes, each pixel in an infrared detector exhibits varying response rates, causing unprocessed raw infrared images to contain non-uniform noise. Additionally, after operating for a period of time, the infrared detector experiences a rise in core temperature, which may cause uneven heating of the shutter and form a pot-cover-shaped image. At the same time, heat radiation from the circuitry can introduce thermal noise and 1/f noise.

Therefore, in addition to optimizing circuit design, infrared imaging algorithms need to be optimized to suppress system noise, eliminate non-uniformity, and improve image quality.

#### 4.1.1. Principle and Limitations of Traditional Correction Algorithm

Traditional non-uniformity correction algorithms mainly include the two-point correction method and the multi-point correction method. The two-point correction method is based on the linear response model of the detector. By capturing the responses of a blackbody at two different temperatures, the gain and offset coefficients of each pixel can be calculated. This method completes non-uniformity correction and features simple structure and low computational complexity. However, it assumes that the detector response is completely linear. In practice, this linear assumption makes it difficult to handle correction errors caused by detector nonlinearity.

The performance gap can be clearly observed from Table 2, where the two-point calibration yields a residual error of ±2%, evidently inferior to the nonlinear correction accuracy of multi-point strategies. Table 2 further quantifies such accuracy–latency trade-off: multi-point calibration improves precision to within ±0.5% but inevitably increases latency up to 20 ms, which imposes constraints on real-time embedded deployment.

The multi-point correction method uses five or more sampling points and cubic or higher-order polynomial fitting. This effectively addresses detector nonlinearity, which reduces correction errors from ±2% to below ±0.5%. However, increasing the number of sampling points causes computational load to grow quadratically. For k = 10, computational load nearly quadruples compared to k = 3. Excess computational burden readily causes processing delays beyond 20 ms, making it difficult to satisfy the 30 fps frame rate and embedded resource constraints required for autonomous power inspection and industrial online temperature measurement. A common mitigation strategy divides the temperature range into 3–5 sub-intervals for 1st- or 2nd-order polynomial fitting, maintaining calibration accuracy while reducing computational load.

#### 4.1.2. Non-Uniformity Correction Technique

Differences in pixel response consistency and uneven lens heating in infrared detectors lead to the ‘center bright and edge dark’ lid effect and global image non-uniformity. Such phenomena seriously affect target recognition accuracy in patrol inspections. Non-uniformity correction technology has evolved from global coarse correction to local accurate suppression through the co-development of calibration methods and scene-adaptive algorithms.

The classical two-point correction method remains the mainstream basic scheme in the industry that obtains correction gain and offset for each pixel through high-temperature and low-temperature blackbody calibration. When applying to most conventional non-uniformity scenes, it can be divided into two implementation paths: blank and non-blank.

Two-point correction with a blank plate uses a motor-controlled blank plate switch to periodically calibrate temperature drift non-uniformity, operating without carrying a blackbody at all times, which is widely used in civil and industrial inspections. However, long-term use can aggravate the lid effect due to uneven baffle heating, and there is a risk of short-term blindness. This makes it unsuitable for patrol scenarios with extremely high continuity requirements, such as military reconnaissance and real-time monitoring of important facilities. The core steps involve calculating and storing correction gain from high and low temperature blackbody acquisition data. During operation, the blank plate closes to acquire images, and the system corrects offset in real time by combining stored gain calculations to complete non-uniformity correction. Two-point correction without a blank plate pre-calibrates detector response parameters for different temperature intervals using a high and low temperature chamber, with these parameters stored in FLASH. However, for outdoor inspection scenarios with wide temperature fluctuations, the method suffers from insufficient calibration precision and limited dynamic range. Experimental results show that the proposed methods effectively improve the edge blackening problem and reduce the area of non-uniformity, but their effect on local suppression of the lid effect remains limited [79]. Correspondingly, Table 2 indicates that the two-point method can only meet basic calibration demands, while the scene-based gradient algorithm achieves over 99% valid imaging area and specially suppresses the pot lid effect.

The correction algorithm based on horizontal and vertical gradients of scene achieves a key breakthrough for local non-uniformity issues such as the lid effect. The core of the correction algorithm lies in using the gradual change characteristics of the lid effect and the abrupt change characteristics of scene information. These two types of information are distinguished through horizontal and vertical gradient calculation. The gradient value of abrupt change areas is large, corresponding to scene details. The gradient value of gradual change areas is small, corresponding to lid noise. The algorithm sets the gradient of abrupt regions to zero by applying an adaptive gating threshold which reconstructs the image while retaining gradual lid information and effectively suppresses the lid effect. In the key optimization process, the algorithm addresses fringe noise caused by gradient zeroing through several steps. Down-sampling with a scale of two first smooths fringe noise. A 5 × 5 median filter then eliminates isolated noise. Horizontal and vertical gradients are calculated, followed by threshold judgment on the sum of squares, with gradients exceeding the threshold set to zero. Image reconstruction and bilinear interpolation are performed, and up-sampling with a scale of two restores the original image size. The algorithm calculates the variance of pixel four-corner regions within a 9 × 9 neighborhood for each frame, then locks and stores the correction parameters.

The algorithm has been implemented on FPGA hardware, meeting the real-time requirements of inspections whose hardware implementation uses RAM to cache 5 × 5 neighborhood data and gradient matrices. FIFO stores anchor points and interpolation results, while DDR3 stores the gain matrix. The entire design is packaged as a general-purpose IP module [80,81]. Actual tests show that the algorithm’s LUT logic occupies only 18 K, with an end-to-end delay of 0.6 ms, which effectively suppresses the pot lid effect in scenarios such as indoor garages and outdoor inspections, increasing the proportion of effective grayscale areas to over 99%. The algorithm demonstrates compatibility with detectors of various resolutions, including 640 × 512 and 1280 × 1024, working with uncooled detectors such as the Arrow RTC6122W and KIP617AC from the Kunming Institute of Physics, as well as mid-wave cooled detectors from the North China Optoelectronics Technology Institute. These measured indices are highly consistent with the parameter records in Table 2, verifying its feasibility for UAV and robot inspection scenarios.

To address the common challenge of non-uniformity correction in different uncooled detectors, a universal on-chip calibration (OCC) method was designed based on the secondary development features of embedded FPGA (eFPGA). This optimizes non-uniformity from the imaging principle level [82]. The core function writes 6-bit calibration data to detector pixels, applying independent bias to each pixel to reduce focal plane array non-uniformity. This complements scene-adaptive algorithms. For example, after calibrating an uncooled detector, 99.77% of pixel grayscale values concentrate within the target range, effectively solving the edge blackout issue. After calibrating the Kunming Institute of Physics KIP617AC detector, non-uniform vertical striping is significantly eliminated, whose hardware optimization uses DDR3 in a two-read, one-write mode to update OCC parameters, caching data through First In, First Out (FIFO) and interacting with DDR3 arbitration IP via the Advanced eXtensible Interface (AXI) protocol [83]. By simply configuring parameters such as the number of calibration frames and pixel rows and columns, this method adapts to most uncooled detectors on the market [84,85].

#### 4.1.3. Random Noise Suppression Technique

The infrared imaging system’s circuit thermal radiation, detector technology defects, and inspection environment interference introduce various random noises. These include thermal noise, 1/f noise, salt-and-pepper noise, and Gaussian white noise. Such noise causes image blurring and detail loss, hindering inspection target extraction. Random noise suppression technology integrates spatial domain and time domain algorithms, ensuring denoising effectiveness while preserving edge details.

Traditional hardware-based denoising algorithms each have their focus in inspection scenarios. However, they face performance bottlenecks and are mainly used for basic denoising in low-noise, simple scenarios:

(1) Median Filter: A nonlinear processing method that replaces the target pixel value with the median of its neighborhood that effectively preserves edge information but does not fully suppress background noise. When the window size increases, detail blurring may occur, which is suitable for initial filtering of isolated noise points during inspections.
(2) Gaussian Filter: Smooths noise through a weighted average based on a normal distribution that effectively suppresses Gaussian white noise but neglects image details, resulting in blurring after filtering. However, this approach is only suitable for removing mild noise, such as in low-noise indoor equipment inspections [82,86].
(3) Non-Local Means Filtering: Uses image patch similarity to perform weighted averaging, which achieves favorable detail preservation. However, it requires substantial computation and consumes significant hardware resources when traversing the entire image, making it difficult to meet real-time inspection requirements and remains largely in theoretical research and non-real-time processing for specific high-precision inspection scenarios.

To address the shortcomings of traditional algorithms, a fusion algorithm based on improved inter-frame filtering and weighted guided filtering has emerged as a main approach. Its core lies in balancing temporal noise suppression and spatial detail preservation, making it well applicable to complex scenarios such as outdoor power inspection and nighttime security monitoring. In terms of algorithm design, temporal denoising uses improved inter-frame filtering. By setting three thresholds—a low threshold (Tl), a high threshold (Th), and a filtering weight threshold (Td)—the algorithm dynamically allocates weights to adjacent frames, which uses inter-frame differences to determine pixel motion status. This allows weighted averaging in static areas to suppress noise while preserving details in moving areas. It effectively prevents motion blur and loss of weak targets and also adapts well to scenarios where moving and stationary targets coexist during inspections. For spatial denoising, weighted guided filtering is employed. Building on classical guided filtering, the algorithm adaptively adjusts the regularization factor based on the image’s dynamic range and local variance. It applies edge-aware weighted processing, assigning higher weights to edge regions and lower weights to flat regions. This approach suppresses spatial noise and reduces halo artifacts while preserving critical details such as equipment cracks.

In terms of hardware optimization, inter-frame filtering uses DDR3 memory to accumulate frame data. This scheme simplifies processing by pseudo-reading DDR3 to use the first frame as the accumulated frame. The design is packaged as an IP module that supports flexible configuration of parameters such as the number of rows and columns and thresholds. The weighted guided filtering adopts a three-channel parallel processing architecture [87]. The data stream is divided into three parallel processes: edge weight computation, guided filter coefficient calculation, and image correction. These processes are synchronized through FIFO timing [88]. Three 14-bit wide, 1280-depth dual-port RAMs implement 3 × 3 neighborhood output. The 11 × 11 neighborhood is achieved through multi-RAM cascading. The final LUT logic occupies 33K, with an end-to-end delay of less than 1 ms [89]. Performance verification indicates that the algorithm remains stable in environments ranging from −10 °C to 40 °C and during work durations of 0 to 8 h. The Blind Image Quality Index (BIQI) image quality index shows an average improvement of 4.80 to 9.31. The denoising effect is superior to median filtering and Gaussian filtering, approaching the current optimal Block-Matching and 3D Filtering (BM3D) algorithm. At the same time, it addresses the challenge of BM3D’s difficulty in real-time hardware implementation. It fully meets the real-time output requirements of 1 k × 1 k resolution at 50 Hz frame rate. In eight different inspection scenarios, including outdoor roads and industrial facilities, it effectively suppresses noise, restores edge details, and exhibits no significant halo artifacts.

### 4.2. Optical System Technology

As the core preprocessing link of infrared imaging, the optical system is responsible for infrared radiation collection, focusing, and optical path control. Its performance directly determines image quality and inspection accuracy. Different types of infrared detectors—cooled, uncooled, and wide-spectrum short-wave infrared—impose distinct and stringent requirements on optical system design. Traditional infrared optical systems face multiple limitations, including single-band constraints, difficulty balancing field of view and accuracy, excessive volume and weight, and poor environmental adaptability. In recent years, deep integration of optical design theory, new material technology, and computational imaging has driven breakthrough progress. Its core goal is to provide high signal-to-noise ratio and high-dimensional raw data for backend signal processing, and it also adapts to the differentiated requirements of cooled, uncooled, and wide-spectrum short-wave infrared detectors while covering multi-scene inspection applications including industrial detection, power operation and maintenance, and security monitoring.

#### 4.2.1. Optical Design for Different Infrared Detectors

Based on the differing optical characteristics of various detectors, the optical system achieves compatibility and adaptation through modular design and parameter adjustment mechanisms.

Uncooled detector adaptation: For uncooled detectors such as the Arrow RTC6122W and Kunming Institute of Physics KIP617AC, the optical system adopts a large field of view and low F-number design (e.g., F 1.0). This matches the low sensitivity characteristics of the detectors, while imaging deviations caused by non-uniformity are compensated through the focusing lens. The lens employs a wide-spectrum antireflection coating covering the long-wave infrared band, reducing optical reflection loss. This optical design synergizes with the detectors on-chip correction (OCC) function, laying a foundation at the optical level for subsequent non-uniformity noise reduction.

Cooled detector adaptation: The medium-wave 1280 × 1024 cooled detector from North China Institute of Optoelectronic Technology requires a low-temperature working environment. The optical system adopts a lightweight design to minimize heat conduction effects on the cooling module. Optical path length is optimized to match the detectors high resolution (1280 × 1024) and high sensitivity characteristics. Lens materials with low thermal expansion coefficients suppress aberration drift caused by temperature changes.

Wide-spectrum short-wave infrared adaptation: For wide-spectrum detectors such as the Sony IMX990 covering 400–1700 nm, the optical system incorporates achromatic design integrating visible light and short-wave infrared band correction. Lenses use InGaAs-compatible materials. Multilayer dielectric coatings achieve high transmittance across the 400–1700 nm band, solving dispersion problems in the short-wave infrared range. This provides chromatic aberration-free optical input for wide-spectrum inspection applications such as silicon chip defect detection and night vision imaging.

#### 4.2.2. Active Illumination and Light Field Encoding

Traditional infrared imaging relies on the targets passive radiation and is easily affected by ambient light and noise. Active illumination and light field encoding technology enhance the target signal strength and dimensions by actively controlling light field parameters, achieving separation of the target and background from an optical perspective.

For low-light and non-visible light inspection scenarios, active illumination technology significantly improves the signal-to-noise ratio through structured light modulation. Its modulated illumination design uses digital micromirror devices (DMD) or spatial light modulators (SLMs) to project coded illumination patterns, such as Hadamard basis or Fourier basis, replacing traditional uniform lighting, and actively encodes target optical field information. For example, in single-pixel imaging systems, a series of modulated light patterns are projected via DMD. A single-pixel detector collects light intensity signals after the modulated light interacts with the target. It is especially suitable for low-light inspection scenarios, such as nighttime power equipment temperature measurements. In broadband short-wave infrared inspections, the broadband illumination adaptation scheme uses an infrared LED array as the active light source. It outputs characteristic bands such as 1050 nm and 1550 nm. These bands pair with detectors operating in high quantum efficiency regions, for example, up to 76% quantum efficiency at 1050 nm, which enhances target reflection signals and significantly improves target recognition in scenarios such as silicon wafer defect detection and underwater target identification [25,90]. The system is also equipped with adaptive lighting control capabilities. Integrated light intensity sensors detect ambient light in inspection environments in real time. The system dynamically adjusts the power and modulation frequency of the active light source. For example, it can increase light source power in rainy or foggy conditions to enhance penetration, and can reduce power in strong light environments to prevent signal saturation, ensuring stability and reliability of target signals in various complex environments.

By using optical encoding methods to embed high-dimensional light field information into two-dimensional acquisition data, the system encodes rich information dimensions at the optical level, including position, phase, polarization, and spectrum. In terms of aperture coding, coded aperture masks replace traditional apertures. Examples include uniformly redundant arrays (URAs) and composite Fresnel zone apertures (CFZAs). These masks modulate and encode the light field at the optical level, compressing target three-dimensional spatial information into a two-dimensional image. For example, in a lensless imaging system, the coded mask encodes the target light field into a specific projection pattern. Polarization coding mainly targets scattering environments such as rain, fog, or turbid water. By integrating polarizers and waveplates into the optical system, the target’s polarization information is acquired. Scattered light and target-reflected light exhibit inherent differences in polarization characteristics. Target-reflected light usually has a higher degree of polarization, while scattered light has a lower degree. By capturing images under orthogonal polarization states, backscattering interference is effectively suppressed at the optical level, significantly improving target clarity in challenging inspection environments such as underwater or foggy conditions. Spectral coding is applied to broadband infrared inspection. Dispersive prisms or gratings serve as spectral encoding devices, decomposing broadband incident light into multiple spectral bands. Combined with the multi-channel output capabilities of detectors, this enables single-frame hyperspectral data acquisition. For instance, in a single-exposure spectral compressive imaging system, coded apertures combine with dispersive devices, allowing simultaneous acquisition of multi-band target information in a single exposure.

#### 4.2.3. Scattering Interference Suppression

Infrared inspection often faces scattering environments such as rain, fog, smoke, and turbid water. The optical system is optimized through physical mechanisms and polarization regulation to suppress scattering interference, improving effective extraction of target signals. The influence of scattered light on imaging quality can be effectively reduced by optimizing optical system parameters. In terms of lens parameter optimization, a small field of view and large depth of field are selected to reduce the angle of scattering light. At the same time, narrow-band filters filter out specific bands with strong scattering, such as the 1450 nm band where water scattering is severe. Characteristic bands with strong target signals are retained, significantly improving the signal-to-noise ratio. In addition, the system uses the optical memory effect to acquire multi-frame speckle images through micro-displacement lenses, and actively accumulates target signals at the optical level and suppresses random scattering noise. In the hardware protection layer, the lens surface adopts super-hydrophobic and anti-fog coatings. These reduce light scattering caused by surface adhesion in rain and fog weather. The interior of optical elements uses low-scattering materials, which reduces scattering loss caused by internal impurities or structural defects and enhances image clarity and environmental adaptability.

Based on the interaction characteristics between polarized light and scattering media, polarization control can effectively suppress scattering interference. The polarization imaging system integrates a polarizer wheel into the optical system, allowing sequential switching between four polarization directions: 0°, 90°, 45°, and 135°, thereby capturing multi-polarization images of the target. The system leverages the difference in polarization between the target and the background. For example, reflected light from metal equipment surfaces has a higher degree of polarization, while scattering backgrounds such as fog have a lower degree. Using polarization differential calculations, it directly separates the target from the scattering background at the optical level. For strong scattering environments such as fire smoke, a circularly polarized light application scheme is adopted, which combines a circularly polarized light source with a circular polarization detector. It takes advantage of the excellent anti-scattering properties of circularly polarized light to reduce the depolarization effect caused by scattered light, significantly enhancing the clarity of target edges [25,91]. Experimental validation shows that compared to traditional linear polarization imaging, circular polarization imaging can improve target contrast by more than 30% in environments such as turbid water. Furthermore, through polarization encoding integration technology, polarization encoding combines with light field encoding. The optical system uses a polarization-sensitive coded aperture to simultaneously acquire the spatial and polarization information of the target. This forms high-dimensional polarization spatial encoded data and enhances the ability to recover and reconstruct target information in complex scattering environments.

#### 4.2.4. Lensless Design

As infrared inspection evolves toward mobile platforms such as drones and robots, optical systems achieve compactness and lightweight design through lensless architecture, while maintaining preprocessing performance and suitability for confined spaces and mobile inspection scenarios.

By using a coded mask to replace traditional lens assemblies, a more compact imaging system with preprocessing capabilities can be achieved. During mask design and optimization, either amplitude-modulated masks or phase-modulated masks are selected. Amplitude-modulated masks include the 2D coded mask used in FlatCam. Phase-modulated masks include the Fresnel zone plate (FZA). The mask is placed close to the detector surface, encoding the target light field into a two-dimensional projection pattern. The mask is fabricated using photolithography and measures only a few millimeters. When paired with the detector, it forms an optical module with thickness at the millimeter scale [90,91]. In terms of light field information extraction, the mask’s positional encoding and depth encoding characteristics embed the target’s spatial position and depth information into the projection data at the optical level. For example, using a compound Fresnel zone aperture (CFZA) mask, its multi-ring design effectively enhances depth resolution. Such a design is particularly suitable for detailed inspections in confined spaces such as power lines and equipment interiors. In addition, for wide-spectrum short-wave infrared inspection applications, lensless systems have been optimized for wide-spectrum adaptation. This is achieved by combining wide-spectrum-compatible masks and detectors. The mask material is selected as quartz glass, paired with an InGaAs detector. This enables wide-spectrum coded imaging in the 400–1700 nm range. While maintaining a highly compact system structure, it fully meets the spectral range and imaging quality requirements of wide-spectrum inspections across various scenarios.

Optical preprocessing without lenses can be achieved using the principle of interference. By obtaining complete complex amplitude information that contains both amplitude and phase information, the system significantly enhances the ability to recognize target details. The interferometric optical design uses a coherent infrared light source such as an infrared laser. The incident light is split into an object beam and a reference beam via a beam splitter. After the object beam illuminates the target, it interferes with the reference beam on the detector surface. This directly forms a hologram that records the targets amplitude and phase information. Such a design does not require traditional lens assemblies, making it compact. The complex amplitude information contained in the hologram provides a rich data foundation. To further improve information quality, the system employs multi-frame illumination enhancement technology. By using multi-angle or multi-wavelength illumination to capture multiple holograms, it effectively suppresses twin image interference and random noise from the optical perspective. For example, in a multi-wavelength digital holography system, switching between infrared light sources of different wavelengths and collecting corresponding holographic data enables great improvement in depth measurement accuracy. This technology is particularly suitable for inspection scenarios requiring high precision, such as industrial defect detection. For common vibration disturbances encountered in outdoor inspections, the system has also been optimized for environmental adaptability. The optical structure uses a flexible interference design to minimize optical path differences caused by vibration. An integrated adaptive optical calibration module monitors and compensates in real time for interference deviations caused by environmental vibrations or temperature fluctuations. This ensures long-term stability and reliability of hologram quality.

### 4.3. System Integration and Intelligent Integration: Multi-Technology Collaborative Enabling Intelligent Inspection

The integration of advanced signal processing and optical systems is not a simple superposition of individual technologies. Instead, it forms an integrated inspection system through deep optical algorithm–hardware cooperation and cross-domain technology fusion. Such a system possesses real-time processing, intelligent decision-making, and multi-scene adaptation capabilities.

#### 4.3.1. Cross-Domain Fusion of Infrared Imaging and AI—Digital Twin and Internet of Things

**Figure 3:**
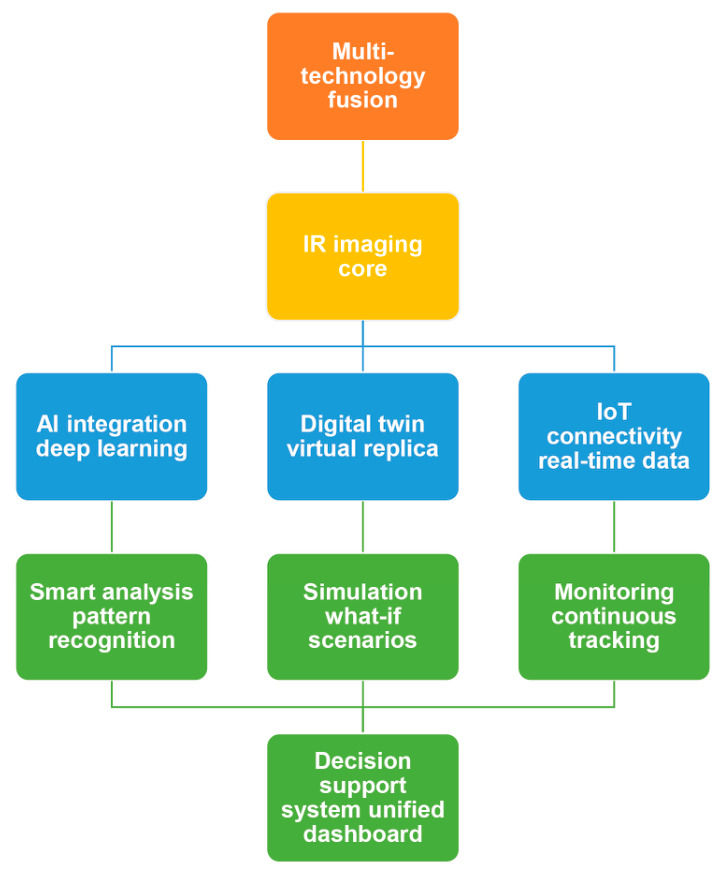
Integrated system architecture for infrared imaging technology fusion. The figure displays the hierarchical framework centered on IR Imaging Core, with multi-technology fusion as the foundation, and three parallel technical branches (AI integration, digital twin, IoT connectivity) converging to a unified Decision Support System for intelligent operation, maintenance, and inspection. Not all application scenarios require all three technical modules simultaneously.

With its core ability of penetrating environmental interference and catching temperature anomalies, infrared imaging technology is deeply fusing with artificial intelligence (AI), digital twins, and the Internet of Things (IoT). This fusion helps break through the bottlenecks of traditional technologies in fields such as new energy operation and maintenance, industrial detection, intelligent construction, and aerospace monitoring. The overall architecture of this integrated system is illustrated in Figure 3.

The cross-domain integration of infrared imaging and AI has achieved a leap from temperature visualization to an intelligent decision-making closed loop. The core value of infrared imaging lies in transforming invisible temperature fields into visual images. However, traditional technology only reaches the level of seeing temperature and struggles to cope with complex environmental interference, massive data processing, and real-time decision-making demands in industrial scenarios. The involvement of AI, empowered by algorithms, brings a cognitive upgrade that enables understanding temperature forming a complete perception–analysis–decision–feedback loop. AI has become the core driving force for the industrial application of infrared imaging technology. The core logic of this integration is based on multimodal data fusion and intelligent analysis. It is not simply stacking algorithms but rather a complete intelligent analysis system. At the data level, AI models integrate infrared thermal imaging data with multi-source data such as visible light, LiDAR, acoustic, and vibration information. Through data alignment, feature extraction, and redundancy elimination, these models calibrate environmental interference affecting a single sensor, including rain, fog, glare, and electromagnetic interference [92], significantly improving the robustness of target recognition.

At the algorithm level, deep learning and machine learning algorithms [90,93] process fused infrared data for target detection, anomaly classification, and trend prediction. These algorithms include convolutional neural networks (CNNs), support vector machines (SVMs), and reinforcement learning (RL). For example, CNNs can automatically identify abnormal heat areas of equipment in infrared images. SVMs can accurately distinguish characteristic differences between normal heating and fault heating. RL can dynamically optimize the sampling frequency and monitoring range of infrared sensors. At the decision-making level, AI converts infrared data into semantic decision results rather than just temperature values or heat maps. For instance, in equipment operation and maintenance scenarios, AI can directly produce clear instructions, such as Equipment bearing temperature abnormal (85 °C, exceeding the threshold by 30%), predicted possible seizure within 2 h, recommend immediate shutdown for inspection, instead of merely indicating that temperature is high [94].

In key technical directions and industrial applications, target intelligent recognition and anomaly classification technology addresses complex industrial backgrounds. Such scenarios include clusters of high-temperature equipment and densely packed pipeline areas. Using techniques such as transfer learning and few-shot learning, the technology solves problems of blurred target features and scarce samples in infrared images [90]. In power inspections, it distinguishes different types of thermal anomalies such as insulator aging, loose conductor joints, and arrester overheating, achieving accuracy of over 95%. In real-time analysis and dynamic optimization technologies, AI algorithms process infrared data streams at the millisecond level. These include real-time stream processing frameworks like Flink and deep learning models [95], which dynamically adjust detection parameters such as temperature measurement range, frame rate, and sampling density. By combining historical data, they build predictive models. For example, in photovoltaic plant operation and maintenance, AI predicts module degradation trends based on infrared temperature distribution of components, optimizing cleaning and maintenance schedules. The drone inspection system of China Resources Powers Anhui branch uses this technology to simultaneously process infrared thermography and visible light data. It automatically identifies cracks in wind turbine blades, which correspond to local overheating, and loose busbar connections, which correspond to localized high temperatures. Compared to manual inspections, efficiency increases more than tenfold. The false alarm rate remains below 0.1%, and maintenance work orders can be generated in real time.

Complex environment adaptation and interference suppression technologies enhance infrared imaging stability in challenging industrial settings. These settings include electromagnetic interference, dust obstruction, and temperature gradient variations. Through adaptive threshold adjustment, image enhancement, and noise suppression algorithms, AI improves performance. For instance, in high-temperature workshops of steel plants, AI dynamically adjusts infrared temperature measurement thresholds based on ambient temperature to avoid false detections. In dust-heavy mining environments, AI uses image restoration algorithms to reconstruct temperature distribution of obscured equipment. By replacing manual interpretation with automated AI analysis, infrared data processing efficiency increases several times. For example, in power grid inspections, what originally required 10 people per day to analyze line infrared data can now be completed by AI in just two hours with more comprehensive coverage. Multimodal fusion and deep learning algorithms improve abnormal temperature detection precision from meter-level to millimeter-level. This enables detection of minor equipment faults such as chip pin cold solder joints and small pipeline leaks with early fault warning further reduces major equipment repair costs. A chemical company, through infrared AI monitoring, successfully prevented a high-temperature leakage incident in a reaction kettle, avoiding direct financial losses of over tens of millions of yuan.

The cross-domain integration of infrared imaging and digital twins has established a physical–digital full lifecycle mapping system. The core of digital twins lies in reproducing physical scenarios through reverse modeling and real-time data access, enabling full lifecycle management of physical objects. Infrared imaging technology provides precise temperature dimension data support for digital twins. This allows virtual models not only to replicate the form and structure of physical objects but also to reflect their operational status in real time, such as thermal distribution and potential faults. This forms a three-in-one digital mapping of form–state–performance [94]. The core integration logic uses temperature data as a link to achieve dynamic synchronization between physical objects and digital models. This mainly consists of three stages. In the data collection and modeling stage, an infrared sensor network collects temperature field data of physical objects such as equipment, buildings, and pipelines. Combined with technologies like laser scanning and BIM, a high-fidelity digital twin model containing temperature attributes is constructed. Each physical unit in the model corresponds to a unique temperature monitoring point, achieving precise binding of temperature data and the geometric model. In the real-time mapping and status update stage, infrared sensors upload temperature data in real time. After preprocessing via edge computing, the data is integrated into the digital twin platform to dynamically update the temperature distribution of the model. For example, in the digital twin model of an industrial kiln, it can display real-time temperature changes in different areas of the kiln wall with accuracy up to ±0.5 °C. In the simulation analysis and decision optimization stage, simulation analyses such as heat conduction simulation and fault propagation simulation are conducted based on the temperature data of the digital twin model. These optimize the operational parameters or maintenance plans of physical objects. For instance, by simulating the temperature field changes in equipment, the equipment lifespan under different operating loads can be predicted, and the optimal load scheduling plan can be formulated.

In key technological directions and industrial applications, the full-lifecycle digital twin of industrial equipment targets devices like fans, motors, and pressure vessels. The digital twin model integrates temperature data from infrared imaging with data on vibration, pressure, current, and other parameters to construct a multi-dimensional state model [93,95,96]. As a key sensing node, infrared imaging connects to edge computing or cloud platforms through the IoT network. This establishes a full-chain system of infrared perception—IoT transmission—cloud and edge applications. It provides large-scale, distributed temperature monitoring solutions for industrial scenarios. The core logic of this integration is to transform infrared sensors into IoT-enabled devices and achieve network collaboration. Specifically, in the terminal IoT transformation stage, infrared sensors undergo lightweight modification. They are integrated with low-power communication modules (such as LoRa, NB-IoT, 5G, and future 6G massive machine-type communication), positioning modules, and edge computing units endowing them with capabilities for data collection, preprocessing, and wireless transmission. They become suited to IIoT resource constraints including low power, low cost, and small size. In the network-layer collaboration stage, infrared sensors connect to industrial networks via IoT communication protocols such as Low-Power Wide-Area Network (LPWAN) (e.g., LoRaWAN) and 5G. This enables long-distance, low-latency transmission of temperature data [97]. To meet the high reliability requirements of industrial scenarios, technologies such as redundant transmission and link backup are used ensuring a data transmission success rate of ≥99.99%. At the application layer integration stage, temperature data is accessed into industrial application systems through the IoT platform. These systems include the Manufacturing Execution System (MES), Supervisory Control and Data Acquisition (SCADA), and operation and maintenance management platforms. The data is fused with information from other sensors to achieve monitoring, early warning, and control functions. For example, when an infrared sensor detects that equipment temperature exceeds the limit, the IoT platform can automatically trigger the SCADA system’s alarm mechanism. It can also coordinate with the Programmable Logic Controller (PLC) to control the equipment to operate under reduced load.

In key technological directions and industrial applications, distributed infrared monitoring technology in the IIoT is deployed in large-scale industrial scenarios such as factory parks, mines, and power grids. Large-scale IoT infrared sensor networks are established, forming a distributed temperature monitoring system. Sensor nodes network through low-power protocols such as LoRaWAN. Edge nodes are responsible for data aggregation and preprocessing. The cloud platform handles data storage, analysis, and application display [92]. For example, a large-scale mines IIoT system has deployed more than 500 IoT-enabled infrared sensors to monitor the temperature of underground equipment, cables, and ventilation ducts. The sensors transmit data to edge nodes on the surface via a LoRaWAN network. The edge nodes analyze the data in real time and issue immediate alerts to the maintenance center via the 5G network once cable temperature exceeds the threshold. The ventilation system is also adjusted to reduce the risk of fire. An electric power company has deployed IoT-enabled infrared sensors on transmission lines. These sensors upload the temperature data of conductors and insulators in real time via the Narrowband Internet of Things (NB-IoT) network. The cloud platform analyzes the data to identify overheating risks. This increases efficiency by five times compared to manual inspections and raises fault detection rates to 98%.

Low-power wide-area infrared monitoring network technology targets remote industrial scenarios such as oil fields and wind farms. LPWAN technologies, such as SigFox and LoRaWAN, are used to build infrared monitoring networks. Sensor nodes are powered by solar batteries with a lifespan of more than five years. The network supports massive node access, with a single gateway capable of connecting to thousands of sensors [97]. This meets the monitoring needs of large-scale industrial scenarios. The IIoT system of a wind farm deploys IoT-based infrared sensors in key parts of each wind turbine. These parts include blades, gearboxes, and generators. Temperature data is transmitted to the wind farm cloud platform through the LoRaWAN network. The platform monitors equipment temperature status in real time and predicts faults in combination with AI algorithms. Infrared sensors also monitor temperature changes in pipelines. When a pipeline leaks, the temperature drops abnormally. The sensor immediately uploads the data, and the cloud platform quickly locates the leak point to reduce crude oil loss. In motor monitoring, infrared sensors monitor winding temperature. Vibration sensors monitor bearing vibration. Pressure sensors monitor lubricating oil pressure. The IoT platform fuses these data to comprehensively judge motor operating status. Raythink Flint Technologys AI thermal imager Xiaorui connects to the factory cloud platform through the IIoT network. It links with vibration and humidity sensors. When infrared detects abnormal motor temperature, it automatically triggers high-frequency collection by the vibration sensor. The IoT platform integrates temperature and vibration data to locate fault types, such as temperature increase caused by bearing wear and increased vibration. It then generates operation and maintenance plans. IoT networks support massive infrared sensor access, enabling comprehensive temperature monitoring in large-scale industrial scenarios and avoids monitoring blind spots. LPWAN technology combined with low-power infrared sensors reduces equipment operating costs. It eliminates the need for frequent battery replacement, making it suitable for remote and unattended scenarios. Infrared monitoring data links with industrial control systems through IoT platforms. This achieves automatic response to faults, such as load reduction, shutdown, and alarm, and reduces manual intervention and improves the safety and stability of industrial systems.

In addition, infrared imaging (perception layer), AI (cognitive layer), digital twins (decision layer), and the Internet of Things (transmission layer) have also achieved collaborative integration [94]. This forms a full-chain intelligent system of perception–cognition–decision–feedback. In the perception layer, the IoT-based infrared sensor network collects temperature data from physical scenes. It combines with sensor data such as visible light and vibration to form multimodal perception data. The transport layer transmits perception data to edge nodes or cloud platforms in real time. It uses IoT protocols such as LPWAN and 5G to ensure low latency and high reliability of data transmission. The cognitive layer analyzes multimodal data through AI algorithms such as convolutional neural network (CNN), SVM, and RL. It identifies temperature anomalies, classifies fault types, and predicts trend changes transforming data into semantic information. In the decision-making layer, the digital twin model integrates AI analysis results. It simulates the effects of different decision-making schemes, such as equipment maintenance plans and operating parameter adjustments, to generate optimal decisions. The feedback layer sends decision-making instructions to execution devices through the IoT network. These devices include robots, PLCs, and O&M systems, enabling fault handling, parameter adjustment, and other operations.

Typical applications include new energy intelligent operation and maintenance systems. In the perception layer, drones equipped with infrared cameras and LiDAR, along with ground-based IoT infrared sensors, collect temperature and morphological data of fans and photovoltaic modules. In the transport layer, the UAV transmits data back in real time through the 5G network. Ground sensors upload data through the LoRaWAN network. Edge nodes aggregate and preprocess the data before connecting to the cloud platform. The cognitive layer uses AI algorithms to identify fan blade cracks, corresponding to infrared local overheating, and photovoltaic module hot spots, corresponding to infrared dotted high temperatures. It classifies fault levels and predicts fault diffusion trends. In the decision-making layer, the digital twin model integrates AI analysis results. It simulates the operating state of blade cracks after repair and optimizes the maintenance time window. It also simulates the impact of photovoltaic module hot spots on power generation efficiency to formulate a cleaning plan. In the feedback layer, O&M personnel receive decision-making instructions through the IoT platform. They remotely control the UAV to accurately locate fault points or dispatch O&M vehicles for disposal. After disposal, they monitor the effect through infrared sensors and update the digital twin model.

The future direction of IIoT is the integration of edge computing, deep learning, and digital twins. The collaboration of these three technologies can solve the three major challenges of real-time processing, resource constraints, and heterogeneous interconnection [97]. The logic is completely consistent with the collaborative architecture mentioned above. IIoT needs to integrate sensing, communication, and data processing technologies. The collaboration of infrared imaging, AI, digital twins, and IoT is a concrete embodiment of this concept. Currently, this integrated system still faces challenges in terms of technology, standards, and cost. On the technology side, IoT transformation of infrared sensors faces the challenge of balancing low power consumption with high performance. Some sensors’ battery life is insufficient for long-term monitoring. AI models lack industrial adaptability, and their generalization ability in complex environments such as high temperature and dust needs improvement. Fault detection accuracy in small-sample scenarios remains low. The update latency of temperature data in digital twin models is high, making millisecond-level synchronization difficult in large-scale scenarios. On the standards side, the interface standards for infrared imaging data with IoT and digital twins are not unified. Interoperability between devices from different manufacturers is poor, and data fusion becomes difficult. On the cost side, the initial investment for large-scale deployment of IoT-enabled infrared sensors and digital twin platforms is high, making it difficult for small and medium-sized enterprises.

In the future, technology will continue to iterate. Infrared sensors will develop toward miniaturization, low power consumption, and high resolution. Combined with energy harvesting technologies such as solar and vibration energy, they will achieve unlimited operation. AI models will evolve toward lightweight, small-sample, and multimodal forms. Edge AI algorithms will be further optimized to reduce dependency on cloud computing. Digital twin models will develop toward real-time, lightweight, and modular forms, which will support rapid deployment and dynamic updates in large-scale scenarios. Application scenarios will expand from industrial operations to aerospace, healthcare, agriculture, and other fields. For example, infrared AI IoT body temperature monitoring will be used in healthcare. Infrared digital twin soil temperature monitoring will be applied in agriculture. When combined with blockchain, this enables trustworthy traceability of temperature data, suitable for food cold chains, pharmaceutical transportation, and other scenarios. Industry standards will also gradually improve. Interface standards for infrared imaging data with IoT and digital twins will be established. This will enhance device interoperability and reduce integration costs.

#### 4.3.2. Embedded Processing and Real-Time Decision-Making in Mobile Platforms

In mobile deployment scenarios for infrared imaging automated inspection, such as drones and ground robots, embedded processing and real-time decision-making are the core supports for achieving autonomous inspection and instant response. The key logic is to use the lightweight computing power of embedded hardware, combined with real-time algorithms and multi-module coordination. It accomplishes critical operations such as infrared data processing, fault detection, and path adjustment in resource-constrained mobile environments without relying on cloud computing, meeting the strict requirements of inspection scenarios for low latency, high reliability, and offline operations. The following sections use typical scenarios such as power inspection and photovoltaic power plant maintenance to elaborate on the technical architecture, core capabilities, and practical applications.

Embedded processing systems are the hardware foundation of mobile inspection platforms. They need to achieve an optimal balance between size, power consumption, and computing power, while supporting complex tasks such as infrared imaging and multi-sensor data fusion and meeting the endurance requirements of drones and the portability needs of robots.

Embedded processing on mobile platforms commonly uses a heterogeneous architecture of FPGA + AI chip + microcontroller (MCU), which achieves efficient processing through task division and collaboration. It also carries out targeted optimizations for the resource constraints of mobile scenarios. This lays a solid foundation for real-time decision-making. FPGAs (field-programmable gate arrays) handle the real-time preprocessing of infrared data, including non-uniformity correction, noise suppression, and image stitching [79]. Their parallel computing capabilities constrain end-to-end processing latency to within 0.6 ms, enabling UAVs to perform real-time, loss-free early warning of conductor joint overheating during high-frame-rate (≥50 Hz) inspections, thereby directly supporting the centimeter-level fault localization and high-speed inspection applications detailed in Section 5.1 [81,87]. For example, in UAV photovoltaic inspections, the FPGA can process 1280 × 1024 resolution infrared data streams in real time. It simultaneously completes hot spot area extraction and coordinate calibration [80]. AI chips (Neural Processing Unit (NPU)/Tensor Processing Unit (TPU)) are responsible for fault semantic recognition and multimodal data fusion. For example, they identify equipment faults such as insulator aging, bearing overheating, and photovoltaic module hot spots in infrared images, achieved using algorithms based on CNN, YOLO, and similar models. Compared with general-purpose processors, AI chips offer higher computing density and lower power consumption. For instance, the embedded NPU on the DJI M3TD drone can perform end-side real-time execution of infrared image acquisition, hot spot recognition, and position calibration. MCUs handle lightweight tasks such as device control and sensor coordination. These tasks include adjusting the exposure time of infrared cameras, synchronizing timestamps of GPS and infrared data, and coordinating motion control with the robot chassis. Their low power consumption and high stability ensure continuous operation of mobile platforms during long-duration inspections.

To adapt to the resource constraints of mobile scenarios, embedded processing systems are optimized in three aspects: algorithm lightweighting, dynamic power management, and multi-interface compatibility with data synchronization [98]. In terms of algorithm lightweighting, complex infrared image processing, and AI recognition algorithms undergo pruning and quantization. For example, the non-uniformity correction algorithm is encapsulated into a general FPGA IP module. Hardware occupancy is reduced through LUT logic reuse and distributed RAM storage [79,80]. The AI fault detection model is quantized to INT8 precision. This reduces computing power requirements by more than 70% while keeping accuracy loss under 3%.

In terms of dynamic power control, the embedded hardware operating mode is dynamically adjusted according to the inspection task phase. For example, during UAV cruising, the AI chip’s computing power is reduced, retaining only the FPGA’s basic data preprocessing. When a suspected fault is detected, the AI chip is triggered to run at full capacity to complete precise fault identification. Low-power chips such as the ARM Cortex-M series MCUs are used alongside power management modules. This keeps the embedded system’s power consumption under 10 W, ensuring UAV flight endurance. Regarding multi-interface compatibility and data synchronization, the embedded system supports various sensors through interfaces like RS232, Ethernet, and SPI. These sensors include infrared cameras, GPS/BeiDou positioning, inertial navigation, and laser obstacle avoidance. Precise multi-source data alignment is achieved via timestamp synchronization. For example, in substation robot inspections, infrared temperature data can be bound with RFID positioning data. This ensures that the fault location error remains less than 5 mm [81].

Real-time decision-making is the core goal of embedded processing systems. Through deep fusion of algorithms and business logic, it realizes the closed loop of data input–analysis–action. This covers key scenarios including fault identification and hierarchical response, path dynamic optimization and multi-module cooperative decision, and offline operation with edge caching capability. In terms of fault identification and hierarchical response, mobile platforms leverage the real-time computing power of embedded processing. They can perform terminal-side semantic understanding of infrared data and fault classification without waiting for cloud feedback. In the real-time fault identification link, the embedded AI chip runs lightweight deep learning models. It analyzes infrared thermal imaging data in real time and identifies equipment abnormality types and severity levels. For example, in power patrol robots, it identifies insulator aging, corresponding to local hot spots, and wire joint looseness, corresponding to point-shaped high temperatures. The identification accuracy rate exceeds 99%, and the false determination rate remains below 0.1%. In photovoltaic power station UAV patrols, the system automatically distinguishes different fault types such as slight hot spots, diode short circuits, and component cracks. It performs hierarchical marking to prioritize operation and maintenance tasks. The embedded system immediately triggers local alarms such as sound and light prompts after fault identification. It returns key information including fault position (GPS coordinates), infrared images, and fault levels to the ground control center through 5G/6G or next-generation wireless local area networks. For emergency faults such as equipment temperature exceeding the threshold by more than 30%, the embedded system directly triggers the generation of operation and maintenance work orders, shortening the fault handling cycle.

In terms of dynamic route optimization and multi-module cooperative decision, real-time decision-making extends beyond fault identification. It must link navigation, obstacle avoidance, equipment control, and other modules to achieve independent optimization of the patrol inspection process. In the path adjustment link based on infrared data, the embedded system automatically triggers path adjustment commands when it identifies a suspected fault area through infrared imaging during patrol. These commands control the UAV or robot to approach the target area for high-definition infrared imaging and multi-angle shooting. For example, during UAV transmission line patrol, if temperature abnormality is detected at a joint, the system automatically adjusts flight attitude and collects infrared data from three different angles to improve fault judgment accuracy. In the multi-sensor cooperative obstacle avoidance and positioning link, the embedded system fuses multi-source data from infrared cameras, laser radar, and inertial navigation. It judges obstacles such as trees and buildings in the patrol environment in real time and adjusts paths to avoid risks. For example, when a substation robot detects obstacles within 0.5 m ahead using laser radar, the embedded system immediately triggers a shutdown command to avoid collision. Combined with RFID positioning and magnetic navigation, the robot achieves positioning accuracy of ±5 mm in narrow channels.

In terms of offline operation and edge caching capability, the embedded system possesses strong offline processing and data caching capabilities. This addresses weak network signal issues often faced by mobile patrol platforms in remote areas. In the offline fault identification link, the infrared feature template library for fault models of equipment such as insulators and transformers is stored in the embedded systems local embedded MultiMediaCard (eMMC) and Secure Digital (SD) card storage. This allows the system to complete infrared data processing and fault identification even when disconnected from the network. In the edge caching and incremental uploading links, the embedded system caches infrared images and fault data during patrol inspections. It adopts an incremental uploading strategy, returning only fault frames and key information. This reduces data transmission volume and saves network bandwidth. For example, a photovoltaic power station patrol UAV caches over 10 GB of infrared data, uploading only fault frames containing hot spots, which account for approximately five percent of the total data volume, to the cloud.

In the practice of typical application scenarios, the embedded processing and real-time decision system demonstrates excellent performance and practical value. It also faces certain technical challenges and defines clear optimization directions. In the UAV photovoltaic power station patrol scenario, the embedded processing architecture adopts FPGA (Xilinx Versal AI Edge series, 7 nm process) + NPU (Huawei Ascend 310) + MCU (STM32H7). The FPGA handles infrared data preprocessing and image stitching. The NPU manages hot spot identification and component degradation trend prediction. The MCU controls UAV flight attitude and camera parameter adjustment. The real-time decision-making capability operates as follows: When the UAV cruises along a preset path, the embedded system processes infrared data streams in real time to identify hot spot faults in photovoltaic modules. Positioning accuracy reaches 1 m. If a large-area hot spot is detected, the system automatically adjusts the flight path to increase patrol density in that area. It simultaneously generates operation and maintenance work orders. In terms of performance metrics, infrared image processing delay remains under 1 ms. Fault identification accuracy exceeds 99 percent. A single patrol cycle can cover over 100 MW of photovoltaic capacity, achieving more than 10 times the efficiency of manual inspection.

In the substation ground robot patrol scenario, the embedded processing architecture centers on FPGAs, complemented by low-power AI chips and MCUs. FPGAs process the fusion and calibration of infrared and visible light images. AI chips identify pointer meter readings and equipment temperature anomalies. MCUs interface with magnetic navigation and RFID positioning modules. The real-time decision-making capability manifests as follows. As the robot autonomously patrols along magnetic marker paths, the embedded system analyzes infrared images in real time. It monitors temperature status across 103 infrared monitoring points. When detecting anomalies such as switchgear temperature exceeding 70 °C, the system immediately triggers local alarms. It adjusts the pan–tilt angle to collect multi-dimensional infrared data for fault verification. Simultaneously, fault information transmits back to the monitoring center through 5G or next-generation wireless communication modules. Temperature measurement error remains within ±2 °C, and equipment identification accuracy exceeds 99.1 percent. A single patrol covers all key devices in the substation, reducing patrol time by 60 percent compared to manual methods. However, the technology still faces three core challenges. First, balancing power consumption and computational performance remains difficult. Mobile platform endurance is highly sensitive to power consumption. Embedded hardware must find equilibrium between increasing computational power to support complex AI algorithms and reducing power consumption to extend operational endurance. Second, real-time synchronization of multimodal data presents significant challenges. Timestamp synchronization errors among infrared images, GPS positioning, inertial navigation, and other multi-source data must be controlled at the millisecond level. Third, algorithm robustness in complex environments remains insufficient. In scenarios involving rain, fog, high temperatures, and strong electromagnetic interference, infrared data remains susceptible to noise interference.

In response to these challenges, optimization directions are clearly defined. Algorithm–hardware co-design leverages deep coupling between customized algorithms and hardware architectures. Fault identification algorithms are optimized in conjunction with FPGA and AI chip architectures. For example, convolution and pooling layers of neural networks map directly into FPGA hardware logic to improve computational efficiency. Edge AI model iterations employ techniques such as few-shot learning and transfer learning to enhance end-side AI model generalization, reducing dependence on large-scale annotated datasets. Model quantization and pruning further lower computational requirements. Enhanced multi-sensor fusion integrates data from infrared imaging, LiDAR, and millimeter-wave radar. This improves fault identification robustness in complex environments. For instance, in rain and fog conditions, LiDAR distance information assists infrared hot spot positioning, reducing scattering interference.

The essence of embedded processing and real-time decision-making in mobile platforms lies in the technological fusion of end-side lightweight computing, real-time algorithms, and module coordination. Through optimized heterogeneous computing architectures and scenario-specific algorithms, infrared imaging automated inspection evolves from manual assistance to autonomous operation. Practical applications in electric power, photovoltaic, chemical, and other industries demonstrate significant benefits. This technology improves patrol inspection efficiency by five to 10 times with fault detection rates exceeding 99 percent. It simultaneously reduces manual operation and maintenance costs and safety risks substantially. Looking forward, continuous improvements in embedded AI chip computing power and low-power technologies will further expand application scenarios. These include consumer electronics, autonomous driving, intelligent medical devices, and other fields. Embedded processing will become the core support for mobile intelligent systems, providing powerful momentum for the intelligent transformation of more industries.

#### 4.3.3. Optical Algorithm–Hardware Cooperative Design of Computational Imaging Driver

The core value of computational imaging lies in breaking the traditional separation mode of optical acquisition + backend processing. It transcends the physical imaging limit through deep cooperative design of optics, algorithms, and hardware. This cooperation is not simple module superposition. Instead, it pursues the goal of end-to-end optimization. Optical modulation adapts to algorithm requirements. Algorithm design matches hardware computational power. Hardware architecture supports real-time processing. This ultimately realizes the closed-loop optimization of physical layer coding + algorithm layer decoding + hardware layer acceleration. Its core logic and implementation pathway require deep analysis combining technical principles, cooperative dimensions, and practical cases.

In traditional imaging systems, optics focuses on clear imaging. Algorithms focus on repairing defects. Hardware serves merely as a data carrier. Performance bottlenecks arise from the independent design of these three components. In terms of target unification, the system discards individual module performance indicators such as optical resolution and hardware computational power. It takes system-level objectives as the core optimization direction. Regarding imaging quality, mean square error (MSE) and peak signal-to-noise ratio (PSNR) serve as core metrics. Optical modulation and algorithmic reconstruction work together to approach theoretical optimal imaging effects under limited hardware resources. In terms of real-time performance, end-to-end processing delay is controlled at the millisecond level, achieved through hardware parallel architecture and lightweight algorithm coordination in scenarios such as patrol inspection and autonomous driving. In terms of resource efficiency, mobile platforms including UAVs and robots benefit from coordination between optical simplification, such as lensless design, and algorithmic compensation. This reduces hardware power consumption and volume while balancing performance and portability. In the aspect of constraint linkage, physical constraints of individual modules are transformed into cross-module cooperative optimization space. Optical constraints such as aperture size and dispersion are compensated by algorithmic decoding, including compressed sensing and phase recovery. For example, coded apertures replace traditional lenses, enabling super-resolution imaging through algorithms. Hardware constraints such as computational power and storage are addressed through optical precoding that reduces data volume. Structured illumination compresses high-dimensional information into two-dimensional acquisition, reducing hardware processing pressure. Algorithm constraints such as real-time requirements and complexity are handled through hardware architecture customization, including FPGA parallel computing and AI chip tensor cores. For example, convolution layers of neural networks are directly mapped onto hardware logic [95].

Three collaborative dimensions constitute the core framework of optical algorithm–hardware deep coupling. In the optical algorithm cooperation layer, the optical system no longer pursues direct imaging. Instead, it provides high-dimensional and redundant original data for the algorithm through encoding and modulation. The algorithm then decodes this data to recover target information. The core cooperation mode includes three types. First, encoding apertures cooperate with compressed sensing. The optical design uses coding masks such as uniform redundant arrays (URAs) and composite Fresnel zone apertures (CFZAs) to replace traditional apertures. These masks compress target spatial information into specific projection patterns. The algorithm employs compressed sensing decoding algorithms. It reconstructs high-resolution images or depth information from limited projection data by leveraging data sparsity. The key advantage lies in reducing optical module thickness to millimeter scale in lensless imaging systems. Second, wavefront coding cooperates with deconvolution algorithms. The optical design introduces cubic phase elements into the optical system. This deliberately introduces controlled distortion to make the point spread function (PSF) insensitive to defocus, thereby expanding the depth of field. The algorithm applies specialized deconvolution algorithms to eliminate optical distortion while preserving target details. This avoids the traditional trade-off between deblurring and preserving details. Practical results demonstrate that this cooperation can expand depth of field by five to 10 times without increasing aperture size, thus avoiding signal-to-noise ratio reduction. It has been successfully applied in infrared patrol inspection and medical imaging scenarios. Third, structured illumination cooperates with super-resolution algorithms. The optical design illuminates the target scene by projecting periodic coding patterns, such as Hadamard patterns and sinusoidal fringes, through digital micromirror devices (DMDs). The algorithm matches the frequency information of the modulation pattern. It breaks through the diffraction limit through phase unwrapping, frequency domain superposition, and related techniques, thereby achieving super-resolution imaging. Compared to pure algorithmic super-resolution, this cooperation reduces hardware computational requirements. It improves real-time performance by more than 30 percent and adapts well to embedded platforms [93].

At the algorithm–hardware co-design level, algorithm design must fully consider the computational characteristics of hardware. Hardware architecture needs to provide customized acceleration for core algorithms. This prevents issues such as the algorithm being complex but the hardware cannot handle it or the hardware having excess computing power but the algorithm is inefficient. This specifically includes three aspects. First, algorithm lightweighting and adaptation to hardware resources target embedded hardware such as FPGA and MCU [90]. Complex algorithms including deep learning and iterative decoding undergo pruning, quantization, and sparsification. For example, quantizing AI recognition models to INT8 precision reduces computing power requirements by 70 percent with less than three percent accuracy loss. For heterogeneous computing architectures combining FPGA and AI chips, algorithms are decomposed into parallel computing modules and serial processing modules. For instance, infrared image preprocessing tasks such as non-uniformity correction and noise suppression are assigned to the FPGA. Fault recognition tasks are assigned to the AI chip, maximizing computing resource utilization. Second, hardware parallel architecture matches algorithm flow through pipeline design. For the entire process of image acquisition, processing, and output, a three-stage FPGA pipeline is designed. This includes data caching, parallel computing, and result output. These stages correspond to algorithm steps of preprocessing, feature extraction, and reconstruction. This reduces data waiting time. Core algorithms such as encoding, decoding, or multimodal fusion are encapsulated into hardware IP cores. For example, the weighted guided filtering algorithm can be fixed in FPGA logic. End-to-end latency remains controllable within 0.6 ms, meeting high-frame-rate requirements of inspection scenarios. Third, storage and computing are optimized collaboratively. For large data volume scenarios such as hyperspectral imaging, the algorithm employs a block processing and incremental decoding strategy. Hardware supports a two-level storage architecture of line buffer and frame storage. For instance, DDR3 caches one line of data. The FPGA processes this data in real time before writing it to the frame buffer, avoiding data congestion. For real-time decision-making scenarios such as UAV inspections, the algorithm prefers an online learning and incremental update mode. Hardware achieves real-time data read and write through dual-port RAM, FIFO, and other devices. This supports dynamic adjustment of algorithm parameters [95].

In the aspect of optical hardware coordination, the optical system design must fully consider the integration characteristics of hardware. The hardware architecture needs to provide accurate support for optical modulation. This achieves seamless connection between physical acquisition and hardware control. This specifically includes three aspects. First, optical parameters synchronize with hardware timing. In terms of timing coordination, the optical shutter speed and integration time are precisely matched with the hardware clock domain. For example, the uncooled detector operates with a 50 MHz clock. The adjustment frequency of the optical electric aperture synchronizes with this clock, avoiding data sampling misalignment. In terms of parameter linkage, the hardware controls optical module parameters in real time through GPIO, I2C, and other interfaces. For example, when the algorithm detects changes in scene brightness, the hardware immediately adjusts the aperture and focal length of the optical lens, implementing adaptive imaging in cooperation with the algorithm. Second, optical simplification cooperates with hardware miniaturization. Lensless or thin-lens designs are adopted. Encoding masks and microlens arrays replace traditional lens groups. Hardware integration design is employed. For example, in the TOMBO system, close fitting of the microlens array and detector achieves an optical module with millimeter-level thickness. The optical system adopts a modular design. It adapts to different detectors, including cooled, uncooled, wide-spectrum, and infrared types, through hardware interfaces. For instance, eFPGA serves as a relay conversion chip. It adapts to timing protocols of different detectors, reducing hardware adaptation costs. Third, optical modulation cooperates with hardware drivers. Hardware provides high-precision driving signals to support optical structured illumination. For example, the mirror-flipping frequency of DMDs, reaching up to kHz levels, is precisely controlled by FPGAs. This enables high-speed encoding projection in coordination with algorithms. The hardware integrates driving modules for optical elements such as polarizer turntables and dispersion prisms. For instance, a stepper motor controls polarizer switching. This allows multi-polarization state data collection in coordination with algorithms to suppress scattering interference [93].

Several typical practice cases verify the implementation value of collaborative design. In the infrared imaging collaborative design for mobile inspection platforms, a large field of view and low F-number lens matches the uncooled detector. The coded aperture pre-codes temperature field information. A scene gradient-based non-uniformity correction algorithm designs gradient noise suppression logic for the optical pot lid effect. The hardware adopts an FPGA three-channel parallel processing architecture. RAM caches 5 × 5 neighborhood data. FIFO synchronizes timing. End-to-end delay reaches 0.6 ms, adapting to the 50 Hz frame rate requirements of UAVs. This ultimately improves the proportion of grayscale effective area to above 99 percent. In the collaborative design of wide-spectrum short-wave infrared imaging systems, apochromatic design covers the 400–1700 nm band. Multilayer dielectric films reduce dispersion. The SLVS differential interface enables high-speed optical signal transmission. The algorithm employs a signal splicing algorithm based on synchronous code search. This solves data stream misalignment issues during optical acquisition. An EMVA 1288 standard calibration algorithm ensures data accuracy [99]. The hardware integrates FPGA with IDELAYE2 and ISERDESE2 primitives for dynamic phase adjustment. DDR3 constructs a two-level cache. USB 3.1 Gen 1 enables high-speed transmission, with a defined upgrade path to USB4 for future high-throughput applications. Real-time processing of 1280 × 1024 resolution is achieved. Final quantum efficiency reaches 76 percent. Dynamic range measures 59.74 dB. The system operates stably in environments ranging from −10 °C to 40 °C for over eight hours. In the co-design of lensless super-resolution imaging, optics employ amplitude-modulated coding masks such as uniform redundant arrays. A compressed sensing decoding algorithm reconstructs super-resolution images from compressed data without iterative optimization. The hardware adopts an MCU and FPGA heterogeneous architecture. The MCU controls mask coding mode switching. The FPGA processes decoding operations in parallel. Power consumption remains within 10 watts. Under a 60 percent volume reduction, imaging resolution approaches that of traditional lens systems. This adapts well to handheld detection equipment.

Currently, collaborative design still faces three core challenges. First, high complexity arises from multi-domain knowledge spanning optics, algorithms, and hardware, making it difficult to unify optimization objectives and constraints. Second, compatibility issues emerge because different detectors and hardware platforms use varying interface protocols, hindering the universality of cooperative schemes. Third, iterative costs remain high. Adjusting a single module requires coordinated re-optimization of other modules, leading to long verification cycles. Corresponding optimization paths include several approaches. Establishing a unified simulation platform enables advance verification of cooperative effects through Zemax 2022 R2.01 for optics, MATLAB R2022b for algorithms, and Vivado 2021.2 for hardware co-simulation, reducing physical iteration. Constructing a modular cooperative framework encapsulates cooperative logic into standardized interfaces. For example, the optical module provides a coding mode library. The algorithm module provides a decoding interface. The hardware module provides an acceleration template. This reduces adaptation costs. Using data-driven optimization builds an optical algorithm–hardware mapping model through deep learning. Data training automatically optimizes cooperative parameters. For instance, a neural network optimizes matching between coded aperture patterns and decoding algorithms.

The essence of computational imaging driven optical algorithm–hardware co-design lies in transforming the limitation of physical imaging into the possibility of cross-module cooperation. In electric power patrol inspection, autonomous driving, medical imaging, and other scenarios, this collaborative design has proven capable of breaking through traditional imaging bottlenecks in resolution, real-time performance, and portability. It has become the core technology direction for next-generation imaging systems. With continuous technological iteration and optimization, its application scenarios will further expand, providing core support for intelligent transformation across more fields.

#### 4.3.4. Multi-Detector Compatible Hardware Integration

Inspection scenarios involve various detector types—cooled, uncooled, and broadband short-wave infrared—each with different interface protocols and driver timing. Traditional post-processing systems suffer from poor adaptability and lead to serious hardware resource waste. By designing a generalized hardware architecture based on FPGA and eFPGA, compatibility and integration of multiple detectors are achieved. Unified deployment of post-processing algorithms becomes possible, greatly enhancing the flexibility and scalability of inspection systems.

The system uses an FPGA as the core processor, paired with DDR3 for large-capacity image caching, FLASH for parameter storage, and eFPGA for secondary development adaptation. This builds a universal hardware platform compatible with both cooled and uncooled detectors [82]. The FPGA selection includes the Xilinx Kintex-7 series, such as the XC7K325 with 326,080 logic cells and 16,020 kb Block RAM, and the low-power Artix-7 series, such as the XC7A75T. The FPGA selection includes the Xilinx Versal AI Edge series (7 nm process node) for high-performance applications and the Artix-7 series, such as the XC7A75T, for cost-sensitive deployments, which would meet the needs for multi-algorithm parallel processing and high-speed data transmission. The hardware architecture encompasses several key modules. Configuration and clock modules provide a 200 MHz clock via PLL division and UART serial debugging. External memory modules use DDR3 for storing frame data and on-chip calibration parameters, while FLASH stores thresholds and calibration parameters. Image acquisition modules handle ADC conversion for cooled detectors through dedicated ADC chips, while uncooled detectors use self-contained ADC conversion. Image display modules support both Cameralink digital display and PAL analog display. In terms of detector compatibility design, the system employs eFPGA as a relay conversion chip. It adapts to different detectors through secondary development of input timing, configuration data, and data integration modules. It also implements on-chip calibration and digital display common modules with fixed design [84]. The system fully supports gated output for both cooled and uncooled detectors. For example, uncooled detectors such as the Arrow RTC6122W and KIP617AC from Kunming Institute of Physics and Technology, as well as the North China Optoelectronic Technology Institutes mid-wave 1280 × 1024 cooled detector, can all have their data gating controlled via the VIO virtual port. The data is then transmitted to the post-processing algorithm module according to a custom data protocol, achieving efficient adaptation and processing.

The software architecture built on the Vivado 2021.2 platform adopts modular development and high-speed bus protocols. This ensures efficient processing and interaction of data from multiple detectors. The core modules include a serial communication module, which receives the host computers’ switch and threshold signals to regulate algorithm performance. A detector driver module provides operational timing and transmits image data via a custom protocol. A dedicated IP for infrared image processing algorithms integrates non-uniformity correction and denoising algorithms. A DDR3 storage module handles data reading and writing through cross-clock-domain FIFO processing and AXI bus arbitration. The AXI4 bus protocol is used for high-speed communication between modules, supporting up to 256 data bursts. Its design separates address, control, and data channels while maintaining independent read and write channels. This caters to high-bandwidth, low-latency post-processing requirements. At the same time, a custom data protocol standardizes the output format of the detectors. For uncooled detectors, a 50 MHz clock and 14-bit data width are used, with each frames effective pixel area taking 920,935 clock cycles. Cooled detectors use an 80 MHz clock, with the effective pixel area taking 1,454,963 clock cycles. Both cases leave ample computing buffer time. Regarding driver generalization, the uncooled detector driver is divided into input timing, configuration data, data integration, on-chip correction, and digital display modules. The on-chip correction and digital display modules remain fixed. Only the input timing and configuration data modules require adjustment to adapt to different models. The cooled detector driver additionally includes an integration control module for controlling integration time and a chip driver module responsible for managing AD5696 voltage configuration and AD7887 temperature reading chips. Its data integration module performs analog-to-digital conversion through the LTC2263 ADC chip.

#### 4.3.5. Broad Spectrum Short-Wave Infrared Signal

The SWIR band offers unique advantages such as silicon material penetration and strong water absorption. Such advantages make it highly demanded in applications like silicon wafer defect detection, night vision inspection, and biomedical imaging. Wide-spectrum SWIR signal post-processing technology achieves wide-spectrum imaging optimization from visible light to short-wave infrared (400–1700 nm) through differential interface analysis, data stitching, and core parameter calibration. This fills the technical gap between traditional infrared and visible light inspections.

Based on the Scalable Low-Voltage Signaling (SLVS) differential output interface characteristics of wide-spectrum short-wave infrared detectors such as the Sony IMX990, a hardware–software collaborative signal parsing scheme was designed. This scheme addresses two main issues: incompatibility between high-speed differential signals and FPGA interfaces, and data misalignment. (1) Hardware interface conversion: Using the MC20901 level shifter, SLVS signals are converted to FPGA-compatible Low-Voltage Differential Signaling (LVDS)_25 signals. This approach is lower in cost and more flexible compared to the traditional Crosslink bridging solution. The SN65LVDS4 differential receiver converts the LVDS clock output from the FPGA into a single-ended clock. This clock is input into the detector to ensure timing synchronization. (2) FPGA signal processing: Dynamic phase adjustment is performed using the Xilinx IDELAYE2 primitive. This compensates for delays caused by PCB traces and devices, ensuring data sampling stability. Serial-to-parallel conversion is completed via the ISERDESE2 primitive. It supports 4-bit parallel output in DDR mode and combines three cycles of data into 12-bit raw pixel data. A synchronization code search strategy is employed to distinguish valid and invalid rows. It detects SAV (Start-of-Active-Video) and EAV (End-of-Active-Video) codes, extracts valid pixel data, and generates row and frame synchronization signals. This provides standardized data for subsequent post-processing [98,100].

The broadband short-wave infrared image data volume is relatively large. With a resolution of 1280 × 1024 and a 12-bit depth, a single frame measures approximately 1.875 MB. To ensure real-time inspection, the system employs an efficient caching and transmission design. In terms of caching, an external 2 Gb DDR3 SDRAM (model MT41K128M16JT) serves as the external frame buffer. Stable timing interaction between the FPGA and the DDR3 is achieved via the Xilinx MIG IP core. The design implements a two-level line buffer–frame storage cache structure. First, an asynchronous FIFO caches one line of pixel data. Once a line is full, a state machine controls its writing into DDR3 for frame-level storage. When reading, entire frames are read from the DDR3 according to the USB transmission timing and recached into the asynchronous FIFO. This solves the issue of clock asynchrony between the FPGA internal clock and the USB chip clock. The transmission interface uses the CYUSB3014 USB 3.1 Gen 1 controller chip (5 Gbps), operating in Slave FIFO mode to achieve high-speed pixel data upload to the computer. For next-generation systems targeting 2030+, migration to USB4 (40 Gbps) is planned to support real-time multi-spectral high-resolution image streaming. The transmission interface uses the CY7C68013A USB2.0 controller chip, operating in Slave FIFO mode to achieve high-speed pixel data upload to the computer. The USB firmware handles chip initialization, endpoint configuration, and communication command parsing. It not only supports remote control of the detectors exposure time, gain, and other imaging parameters from the host computer but also receives commands from the host and forwards them to the FPGA. This enables real-time dynamic adjustment of post-processing algorithm parameters, ensuring that the entire system operates stably and reliably in applications with large data volumes and high real-time requirements.

To ensure the accuracy and reliability of data output from the broadband short-wave infrared imaging system in scenarios such as industrial defect detection and nighttime inspections, a complete parameter measurement platform was established based on the European Machine Vision Association 1288 Standard (EMVA1288) [99]. Key performance indicators were systematically calibrated. The measured results of core parameters show that the system exhibits good response within the 400–1700 nm broadband. The responsivity at three characteristic wavelengths of 400 nm, 1050 nm, and 1550 nm is 0.0735 DN/photon, 0.1175 DN/photon, and 0.1089 DN/photon, respectively. These correspond to quantum efficiencies of 52%, 76%, and 60%. The dynamic range reaches 59.7462 dB, fully meeting the stringent requirements for image detail richness and detection sensitivity in practical applications. In terms of environmental adaptability verification, tests were conducted by simulating typical inspection environments ranging from −10 °C to 40 °C using high and low-temperature chambers. The results indicate that the system operates stably under different temperature conditions. Non-uniformity and noise levels remain within reasonable ranges. Even after prolonged operation of 8 h, the embedded post-processing algorithms effectively suppress noise caused by integration time accumulation or thermal drift. This ensures long-term stability of output image quality. Overall, Table 3 compares the image quality performance in terms of PSNR (peak signal-to-noise ratio) and SSIM (Structural Similarity Index) for different signal processing algorithms under typical complex inspection scenarios. It intuitively quantifies the comprehensive robustness of each algorithm against fog, EMI (electromagnetic interference) and long-distance interference, providing a reliable basis for practical algorithm deployment in power engineering.

## 5. Typical Applications

**Figure 4:**
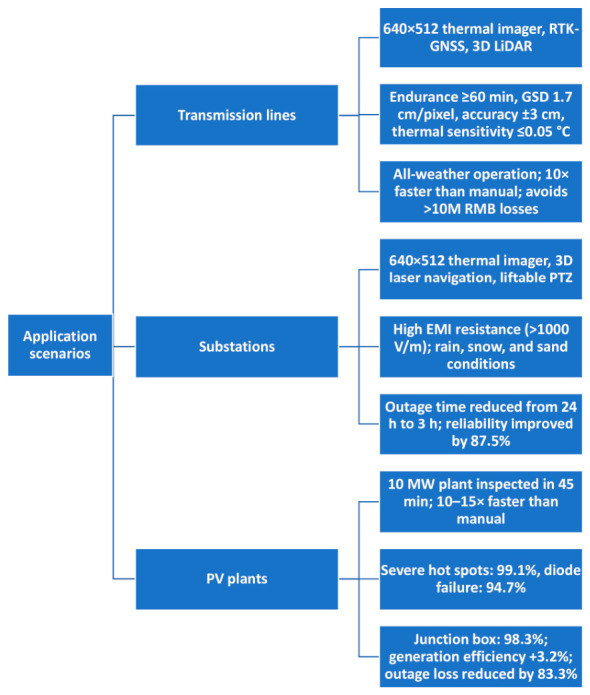
Application technology mapping for power inspection. The figure displays the hierarchical mapping from power inspection application scenarios (transmission lines, substations, PV plants) to key performance metrics, with inspection platforms and core technologies as the intermediate layers.

After long-term operation, transformer equipment in power systems is prone to thermal anomalies and failures, resulting from aging, load changes, environmental influences, and other factors. Traditional manual inspection and periodic maintenance methods have limitations in timeliness and accuracy, necessitating a more intelligent and automated approach for real-time transformer monitoring. Inspection plans for different scenarios vary in technical configuration, performance, and economic benefits. Table 4 summarizes and compares the key parameters of these plans. Infrared thermal imaging technology, which features non-contact operation and online monitoring capabilities is widely used for temperature monitoring of power equipment. For the three core scenarios of transmission lines, substations, and photovoltaic power stations, this review develops a layered and adaptable inspection technology system. As shown in Figure 4, the key technical configurations, performance parameters, and application benefits of the inspection systems across the above three scenarios provide a comprehensive overview of the differentiated technical solutions and their practical applications.

### 5.1. Inspection of UAV Transmission Line

**Figure 5:**
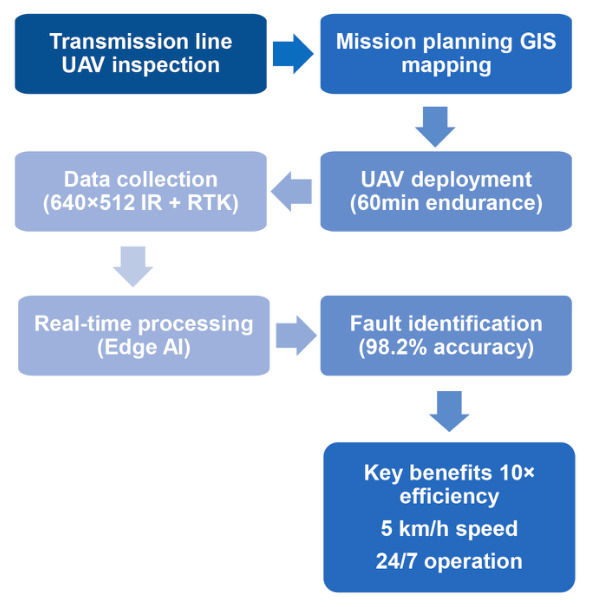
Workflow and performance metrics of UAV-based transmission line inspection with edge AI integration. This schematic outlines the end-to-end workflow of UAV inspection for power transmission lines, integrating geospatial planning, high-performance data acquisition, edge computing, and core performance outcomes.

Power transmission lines are widely distributed across complex terrains and exposed to extreme weather conditions for extended periods, which easily induces fault risks such as loose conductor joints and contaminated insulators and may further cause large-scale power outages. Drone infrared inspection technology offers advantages of high mobility and wide coverage, becoming the core solution for autonomous transmission line inspection. Figure 5 illustrates the complete technical process. The technology implementation revolves around the entire process of equipment integration, route planning, data collection, and intelligent analysis, ensuring accurate and efficient inspection execution.

**Figure 6:**
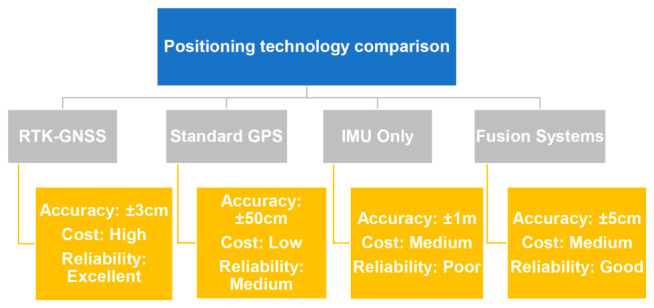
Comparison of positioning technologies for power inspection. The figure displays the hierarchical comparison of four positioning technologies (RTK-GNSS, standard GPS, IMU Only, fusion systems) in terms of accuracy, cost, and reliability for power inspection scenarios.

The technological maturity and miniaturization of uncooled microbolometers (Section 3), together with advances in FPGA-based real-time non-uniformity correction algorithms (Section 4.1), have laid the engineering foundation for the miniaturization, high frame rate, and precise temperature measurement capabilities of the UAV inspection system presented here. The first technical step is integrating the drone with the sensing equipment. A multirotor drone is selected with endurance of at least 60 min and payload of at least 2 kg, primarily equipped with a high-resolution infrared thermal imager. The imager has a resolution no less than 640 × 512 pixels and thermal sensitivity no greater than 0.05 °C [109,110]. The system simultaneously integrates an RTK-GNSS positioning module, 3D LiDAR, and an inertial navigation unit [111]. To maintain centimeter-level accuracy and stability when satellite signals are blocked, the system fuses RTK-GNSS with inertial navigation. Figure 6 compares the performance of different positioning technologies. As shown in the figure, the RTK-GNSS inertial navigation fusion scheme achieves the best overall performance in accuracy and reliability, making it the core positioning technology for inspections in complex terrains. RTK-GNSS ensures centimeter-level positioning accuracy of ±3 cm. LiDAR provides real-time obstacle avoidance. Inertial navigation maintains flight stability when satellite signals are blocked. To adapt to strong electromagnetic environments, the drone’s casing features electromagnetic shielding design [112]. The sensor interface is wrapped with a copper foil shielding layer, reducing interference from environments with electric field strength exceeding 1000 V/m.

The path planning process uses a preset with dynamic adjustment model. Based on the GIS geographic information model of the transmission line, a grid-like inspection area is divided. A preset flight path ensures that the overlap rate of adjacent flight swaths reaches at least 80 percent. Flight altitude is dynamically adjusted according to the infrared thermal imager parameters, keeping the ground sample distance (GSD) at 1.7 cm per pixel [109].

For complex sections such as cross-river, cross-sea, and mountainous areas, a segmented inspection and data stitching strategy is adopted [113]. After the drone autonomously completes each section inspection, the backend system automatically stitches the infrared thermal imaging data together, generating a complete line inspection report.

The data collection and analysis phase focuses on accuracy and efficiency. The infrared thermal imager continuously captures images at a frame rate of 20 frames per second, simultaneously recording temperature data, GPS location, and environmental parameters including temperature and humidity. Through a 5G edge computing architecture, data is quickly transmitted and initially filtered. Only images showing temperature anomalies with a temperature difference of at least 5 °C are uploaded to the backend [114,115], reducing transmission bandwidth usage. The intelligent analysis module employs an image fusion and feature-matching algorithm, overlaying infrared thermal images with visible light images to extract areas of temperature anomalies. Combined with a preset fault feature database, it automatically identifies fault types. The database includes examples such as heating at wire joints, hot spots on insulators, and high temperatures in fittings. Machine learning algorithms distinguish fault levels into three categories: minor faults correspond to temperature differences of 5 to 10 °C. Moderate faults correspond to 10 to 15 °C, and severe faults exceed 15 °C. The system generates diagnostic reports containing fault location, temperature data, and risk level [115].

**Figure 7:**
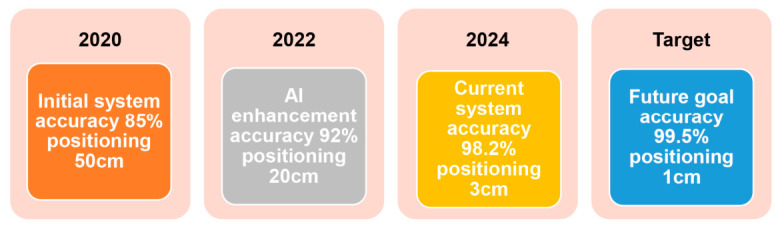
Evolution of UAV-based power inspection system performance (2020–future). This timeline illustrates the progressive improvement in the accuracy and positioning precision of an intelligent power inspection system, driven by iterative technological upgrades and AI integration.

The effectiveness of this technology has been fully verified by experimental data. In a comparative inspection experiment on a 220 kV mountainous transmission line, the UAV infrared inspection achieved an inspection speed of 5 km per hour. This is 10 times higher than manual inspection at 0.5 km per hour [109,116]. Positioning accuracy experiments demonstrate that the RTK-GNSS and inertial navigation fusion scheme achieves fault positioning error of less than 5 cm, representing a 90 percent improvement in accuracy compared to traditional GPS positioning with ±50 cm error. In all-weather adaptability tests conducted in foggy conditions with visibility below 50 m and strong winds up to 15 m per second, the fault detection rate remained above 90 percent. Manual inspections could not conduct effective detection under these conditions. From the perspective of technological iteration over time, Figure 7 illustrates the continuous improvement in system positioning and recognition accuracy, which clearly shows the performance leap from the initial system in early 2020 to the current system in 2024. It also clarifies the future goal of achieving 1 cm positioning accuracy and a 99.5 percent recognition rate.

### 5.2. Substation Inspection Robot

**Figure 8:**
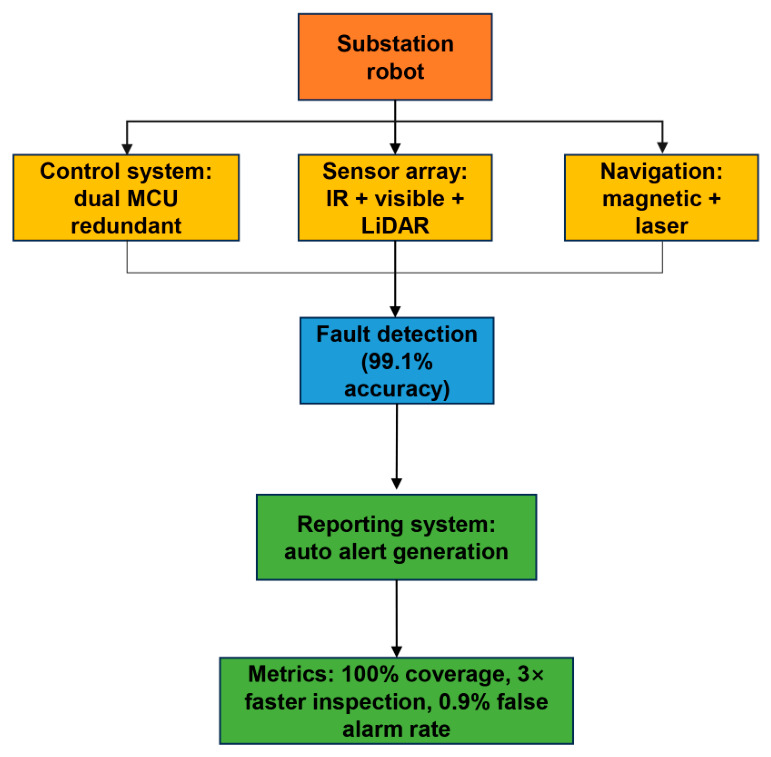
Architecture and performance of an autonomous substation inspection robot. This diagram illustrates the modular system architecture of a substation inspection robot, highlighting its core subsystems, fault detection capabilities, reporting mechanisms, and key performance metrics.

Substation equipment is densely arranged and operates in strong electromagnetic environments, where traditional manual inspection suffers from low efficiency, high risk, and high omission rates. With the deep fusion of infrared imaging and autonomous patrol robots, a full-process inspection system has been established, following the framework of autonomous navigation, accurate temperature measurement, intelligent identification, and real-time early warning. The technology enables equipment-adaptive focusing, path optimization, and fault diagnosis, meeting the requirements for high-precision and full-coverage inspection of substations. Figure 8 shows the technical implementation process.

The strong electromagnetic interference environment in substations imposes stringent robustness requirements on inspection systems. By deploying the scene-based horizontal–vertical gradient correction and multi-parameter joint denoising algorithms discussed in Section 4.1.2, the system effectively suppresses image non-uniformity and random noise induced by electromagnetic induction and elevated background radiation, thereby ensuring the accuracy of subsequent fault identification. The core of the technological implementation lies in the hardware configuration and environmental adaptation of the inspection robot. The robot is equipped with a high-definition infrared thermal imager with a resolution of 640 × 512 pixels and thermal sensitivity of 0.05 °C, and it also integrates a visible light camera. The system features a liftable gimbal with a lifting range of 0.5 to 3 m and a 360° rotation mechanism [117,118], ensuring comprehensive shooting of key equipment such as transformers, GIS devices, and insulators. The navigation system uses an integrated solution combining 3D laser navigation and magnetic navigation [119]. A 3D environment map of the substation’s interior and exterior is pre-built. The robot uses LiDAR to match environmental features in real time, with the magnetic navigation module assisting in positioning, achieving a positioning accuracy of ±2 cm [118]. To address strong electromagnetic interference in substations, the robot’s main control unit features a dual microcontroller unit (MCU) redundant design. Sensor cables are made of shielded materials, ensuring stable operation in environments with electric field strength exceeding 1000 V per meter. The enclosure has an IP67 protection rating, making it suitable for harsh weather conditions including rain, snow, and dust [117,120].

The technical implementation of the inspection process is divided into three main stages: route planning, point inspection, and data processing. In the route planning stage, inspection priorities are determined based on equipment density and importance and high-density inspection points are established in key areas such as transformers and GIS equipment [121,122]. Indoor equipment including switchgear and control panels are planned with sequential inspection routes, ensuring no visual blind spots. During fixed-point inspections, the robot stops 5 m in front of each piece of equipment for 2 to 3 min. The infrared thermal imager focuses on key parts including transformer oil tanks, GIS equipment enclosures, and insulator strings. Temperature data is collected at a frame rate of 15 frames per second. Parameters such as equipment load current and ambient temperature are simultaneously recorded. A temperature calibration model corrects environmental interference: *T*_correction = *T*_measured + *k* × (*T*_ambient − *T*_reference) [15,123]. In the data processing stage, a feature extraction and template-matching algorithm extracts temperature anomaly areas from infrared thermal images. These areas are compared with a preset fault feature library. The library includes examples such as transformer tap changer hot spots, GIS equipment local overheating, and insulator hot spots, allowing the system to automatically identify fault types. An alert is triggered when the temperature difference exceeds thresholds: core equipment triggers at ≥8 °C, and ordinary equipment triggers at ≥10 °C [124].

For different indoor and outdoor equipment, a differentiated inspection strategy is employed. For high-altitude outdoor equipment such as insulator strings and bushings, the robot adjusts the infrared thermal imager angle using a telescopic robotic arm. The robot adjusts the infrared thermal imager angle using a telescopic robotic arm with a range of 0 to 1.5 m, capturing the umbrella skirt area up close and identifies hot spots caused by dirt or damage. For indoor equipment such as switchgear and battery banks, the robot uses a miniature infrared thermal imager to access narrow spaces [122]. It captures temperature signals of busbar joints and individual battery cells. In battery pack testing, a temperature difference of ≤2 °C is considered normal, while a difference exceeding 5 °C is determined to be a fault.

Experimental data verified the effectiveness of this technology. Data from a six-month trial operation at a 500 kV ultra-high-voltage substation shows significant results. The inspection robot achieved 100 percent coverage in infrared monitoring. It issued a total of 114 thermal fault warnings. Correct warnings numbered 113, yielding a warning accuracy of 99.1 percent. The false alarm rate was only 0.9 percent. Detailed equipment fault identification data demonstrates high accuracy. Transformer fault identification accuracy reached 98.7 percent. GIS equipment fault identification accuracy reached 97.6 percent. Isolating switch fault identification accuracy reached 98.2 percent. Compared with manual inspections, performance improvements are substantial. The average detection time for latent thermal faults decreased from 48 h to 2 h. The fault miss rate decreased from 8.3 percent to 1.2 percent. A single inspection requires 60 min, with three scheduled inspections per day, which is three times more efficient than manual inspections (which require 180 min per inspection and are conducted once daily) [120]. An application case from the Changping Shangmei Power Substation demonstrates practical benefits. After deployment, outage time from thermal failures dropped from 24 to 3 h annually, improving supply reliability by 87.5% and reducing direct economic losses by over 5 million yuan.

### 5.3. Fault Diagnosis of Photovoltaic Power Station

**Figure 9:**
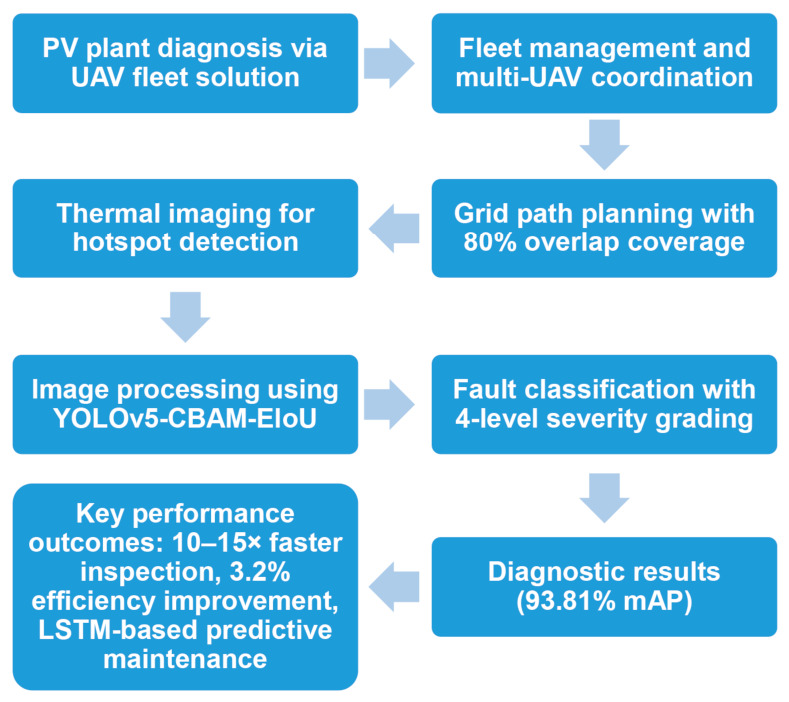
Workflow of UAV fleet-based PV plant diagnosis and efficiency enhancement. This diagram outlines the end-to-end workflow of an intelligent PV plant diagnosis system using a UAV fleet, from mission planning to efficiency gains, with key technical modules and performance metrics.

Photovoltaic power station components are exposed outdoors for extended periods and are susceptible to high temperatures, shading, and other adverse factors, which frequently induce faults such as hot spots and diode failures and further reduce power generation efficiency. Intelligent UAV infrared patrol technology addresses these challenges. It follows the framework of full coverage patrol, accurate identification, and trend prediction. It has become the core means of fault diagnosis in photovoltaic power stations. It effectively solves the pain points of low efficiency and incomplete coverage found in traditional manual inspections. Figure 9 illustrates the complete technical process.

The improved inter-frame and weighted guided filtering fusion denoising algorithm (Section 4.1.3), along with the lightweight YOLOv5 object detection network (Section 4.3.2), provide the algorithmic backbone for the high-speed inspection and real-time hot spot fault identification in photovoltaic plants described in this section. The core of the technical implementation is the integrated optimization of the drone and the inspection system. A multirotor drone is selected (endurance ≥ 60 min, payload ≥ 2 kg). It is equipped with a high-resolution infrared thermal imager (resolution 640 × 512 pixels, thermal sensitivity 0.05 °C), a GPS positioning module, and a LiDAR. To ensure full inspection coverage, a grid path planning algorithm is used. Inspection grids are divided according to the distribution of photovoltaic module arrays, and the drone flies sequentially along these grids. The overlap rate of adjacent flight paths is maintained at 80 percent or higher [125]. Flight altitude is dynamically adjusted according to module height, achieving a ground sample distance of 1.7 cm per pixel, which meets the detection requirement of 5 × 5 pixels per 15 × 15 cm photovoltaic cell. LiDAR measures the distance between the drone and the modules in real time, automatically adjusting flight altitude to avoid changes in shooting distance caused by terrain undulations [126].

The data collection and fault identification process focus on accuracy and intelligence. The infrared thermal imager continuously captures images at a frame rate of 15 frames per second, simultaneously recording module temperature, GPS location, ambient temperature, and other data. This information is transmitted to the ground station via 5G and future 6G dual-mode communication [8]. For large-scale photovoltaic power plants, edge computing technology performs initial filtering on the drone side. Only temperature anomaly images (temperature difference ≥10 °C) are retained, reducing data transmission volume. Fault detection uses an improved You Only Look Once version 5 (YOLOv5) algorithm, integrating a Convolutional Block Attention Module (CBAM) and an Efficient Intersection over Union loss function (EIoU). Infrared thermal images are first preprocessed with denoising and contrast enhancement. Temperature anomaly areas are then extracted and automatically classified according to the established fault feature library. Fault types include hot spots (localized high-temperature areas, ΔT = 10–50 °C), diode failures (strip-shaped hot areas, ΔT ≥ 20 °C) [127], component breakage (dispersed hot spots), and junction box heating (concentrated hot spots, ΔT ≥ 20 °C). The algorithm calculates parameters such as the fault area and peak temperature to assess the severity of the fault and generates precise coordinates and diagnostic reports for the faulty components [128].

System-level fault diagnosis and long-term trend prediction technologies further enhance maintenance effectiveness. For photovoltaic string connectors, drones use a close-range fixed-point photography mode. They fly to approximately 0.5 to 1 m near the connectors to focus and shoot, identifying hot spots caused by looseness [126]. For inverters, aerial inspections are conducted around the enclosure focusing on heat sink areas. The temperature distribution of different power modules is analyzed. Normal modules exhibit temperature differences of ≤5 °C, while faulty modules show temperature differences of ≥15 °C. The multi-drone collaborative inspection framework is suitable for large-scale power plants of 100 MW or above. The main drone is responsible for task allocation and data aggregation. Subordinate drones carry out inspections synchronously by area. A dynamic task scheduling algorithm balances the workload to avoid duplication or omissions. Long-term trend forecasting is based on six consecutive months of infrared inspection data. Component temperature variation models are established using a Long Short-Term Memory (LSTM) algorithm [8]. When a component’s monthly temperature increase rate reaches 3 °C or higher, it is marked as a high-risk hazard, enabling proactive replacement.

**Figure 10:**
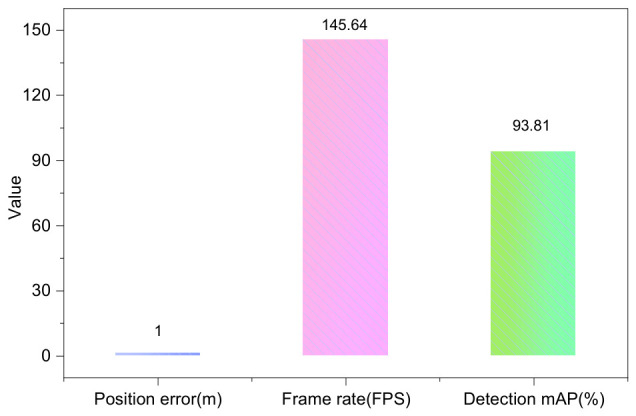
Overall performance scores of the intelligent inspection system in key dimensions. This radar chart (or multi-dimensional bar chart) illustrates the system’s comprehensive performance across four critical operational dimensions, demonstrating its efficiency and stability in real-world inspection scenarios.

Experimental data fully validates the effectiveness of this technology. In an inspection experiment of a 1.56-hectare photovoltaic power station (10 MW), the drone completed collection of 321 infrared images of the entire station in just 45 min. The improved YOLOv5 algorithm achieved a hot spot fault detection precision of 96.87 percent, a recall rate of 89.71 percent, a mean average precision of 93.81 percent, and a frame rate of 145.64, which significantly outperformed traditional algorithms such as Faster Region-based convolutional neural networks (Faster R-CNN) (mAP 86. 03%, FPS 78.36). In terms of real-time operational performance, Figure 10 shows the system’s performance on key indicators. As shown in the figure, the system’s overall performance scores in the four key dimensions of positioning error, processing delay, frame rate, and detection confidence consistently remain above 85 points, which confirms the algorithm’s efficiency and stability in real inspection scenarios. The identification accuracy for different fault types was as follows: minor hot spots, 95.2%, severe hot spots, 99.1%, diode failure, 94.7%, and junction box overheating, 98.3%. Application data from a 100 MW photovoltaic power station demonstrated significant benefits. Drone infrared inspection was 10 to 15 times more efficient than manual inspection [128]. Fault location error remained within 1 m. Through long-term trend prediction, 23 potentially faulty components were replaced in advance, increasing the power station’s generation efficiency by 3.2 percent. The average annual downtime caused by faults was reduced from 72 h to 12 h. Power outage losses decreased by 83.3 percent. In harsh environment adaptability tests conducted under high temperature and strong sunlight (40 °C) and foggy conditions (visibility below 50 m), the fault recognition rate remained above 90 percent, meeting the requirements for all-weather inspections [125].

## 6. Challenges and Perspective

### 6.1. Material: New Sensing Materials and Device Fabrication

The material foundation for next-generation infrared detection is shifting from performance pursuit alone to a balanced co-optimization of sensitivity, environmental robustness, manufacturability, and system-level integration. Early design assumptions—such as relying solely on pristine material superiority—have proven inadequate for the complex, long-term unattended operation required in power grids. Future progress will be driven by synergistic strategies: heterostructure engineering, hybrid integration, and application-specific customization.

#### 6.1.1. Two-Dimensional Material

Novel two-dimensional material systems deliver targeted performance improvements for infrared detection under harsh outdoor grid conditions, showing unique application potential for portable inspection terminals and long-term unattended monitoring. Surface passivation, heterostructure engineering, and optimized packaging strategies enhance the environmental robustness of two-dimensional devices, and improve resistance to temperature fluctuation, humidity erosion and complex atmospheric interference in field inspection. For instance, atomic layer deposition modification greatly improves the air stability of key two-dimensional materials [129], while 2D–2D van der Waals heterojunction design effectively inhibits material aging and optimizes the transmission of photogenerated carriers, which strengthens the identification ability of weak thermal defect signals in substations and transmission lines [130].

Two-dimensional devices can realize customized spectral matching with typical thermal radiation features of power equipment, enabling accurate response to mid-infrared and long-wave infrared signals induced by equipment overheating, insulation dampness and component aging, thereby strengthening the quantitative diagnosis capability of latent grid faults [129,131,132]. Composite integration with traditional semiconductors and optical structures further compensates for the performance deficiencies of single materials, achieving coordinated improvement in response speed and detection sensitivity. Such integrated devices possess good platform compatibility and are suitable for lightweight loading of UAVs and inspection robots.

Despite these advances, several practical bottlenecks remain for deployment. Key challenges include poor uniformity in large-area films, low optical absorption of atomically thin materials, and high array fabrication costs. Beyond such universal limitations, power grid scenarios pose unique threats: strong power frequency electromagnetic fields and high-frequency interference induced by partial discharge readily disturb carrier transport and degrade signal stability. Long-term exposure to outdoor pollution, icing, and sandstorm conditions also accelerates device aging. Future breakthroughs will rely on anti-electromagnetic interference design at the device level (e.g., shielded packaging and differential readout) and harsh environment reinforcement (e.g., self-cleaning surfaces and passive thermal management), moving from laboratory demonstrations to grid-proven reliability.

#### 6.1.2. Quantum Point Technology

Quantum dot-integrated devices exhibit prominent comprehensive advantages in low-cost mass manufacturing, flexible forming and wide-spectrum perception, which match the development demands of large-scale distributed power inspection systems. Narrow-bandgap quantum dots cover the 3–15 μm infrared band and accurately detects weak thermal radiation from overheated cables, switches, and distribution equipment [131,132], effectively remedying the insufficient weak signal detection capability of traditional uncooled detectors and improves the early warning level of subtle potential faults in power facilities.

The hybrid structure of quantum dots and two-dimensional materials optimizes photoelectric conversion and carrier transmission efficiency, while enhancing dynamic response and weak defect recognition capability, satisfying the requirement for real-time perception of transient electrical faults [131]. Relying on excellent solution processability, quantum dot devices can be fabricated into flexible curved arrays through low-cost printing processes, adapting to the special installation surfaces of transmission towers and transformer shells, and expanding the full-coverage inspection range of intelligent inspection equipment.

Poor stability against oxygen and moisture remains the primary challenge for long-term outdoor operation. Although ligand modification and encapsulation technologies have significantly improved the environmental adaptability of quantum dot detectors [131,132], long-term aging resistance and operational reliability under extreme temperature and humidity conditions still lack long-term verification. In actual high-voltage grid environments, flexible quantum dot arrays also suffer from mechanical fatigue under low-temperature icing and surface corrosion caused by industrial contamination. Severe electromagnetic coupling interference further affects long-term operational stability and batch consistency, becoming a critical constraint for large-scale on-site deployment.

#### 6.1.3. Metasurface Integration

Metasurface optical manipulation enables miniaturization, lightweight design, and functional integration for infrared inspection systems, which can compensate for the shortcomings of traditional detection systems such as bulky volume, single spectral response and low light utilization efficiency. Customized metasurface design integrates infrared focusing, polarization screening and band filtering functions, replacing discrete and heavy optical components, significantly reducing the volume, weight, and power consumption of detection terminals. It is highly applicable to UAV inspection platforms with limited payload and endurance constraints [133].

Reconfigurable metasurfaces with dynamic spectral tuning functions can flexibly switch the operating band according to different fault types, including conductor overheating and insulator deterioration [133,134,135], supporting multi-dimensional collaborative detection on a single device and greatly improves the overall inspection efficiency and multi-scene adaptation capability of autonomous inspection systems. The monolithic integration of metasurface and sensitive layers can produce local light field enhancement effects, improve the light absorption efficiency of sensing materials, and further promote imaging definition and ultra-weak defect detection performance [136].

In future research, low-cost and large-area micro-nano processing technology will be the key breakthrough direction [134,135]. Reducing the manufacturing and application cost of metasurface composite detectors is conducive to promoting their large-scale popularization in intelligent power inspection, and laying a foundation for the upgrading and iteration of high-performance integrated infrared inspection terminals. From the perspective of grid application, dust deposition, surface contamination and insulator icing will distort optical modulation characteristics and introduce detection errors. Moreover, multispectral inspection can cause optical crosstalk among infrared, ultraviolet, and visible imaging, necessitating structural optimization and multi-band decoupling.

#### 6.1.4. Others

In addition to low-dimensional materials, various emerging functional materials have formed a diversified infrared detection system that can meet differentiated high-precision detection requirements under special power grid operating conditions, while still facing inherent application limitations. Topological insulators feature ultra-low noise and ultrafast photoelectric response, and are suitable for capturing weak transient signals such as partial discharge of high-voltage electrical equipment. Nevertheless, complicated preparation procedures and high integration costs hinder their large-scale engineering application in power industries [137].

Perovskite materials offer high quantum detection efficiency and low-cost fabrication advantages, yet their poor thermal and moisture stability cannot adapt to long-term harsh outdoor conditions, restricting their deployment in unattended long-term inspection [137]. High-temperature superconducting detectors deliver ultra-high detection sensitivity, which can meet the ultra-precise monitoring needs of key power equipment. With the gradual miniaturization and cost reduction in refrigeration technology, such high-end detectors are expected to be applied in core hub substations in the future, but high operational costs and complex electromagnetic working conditions remain the main constraint for wide-scale application in power inspection.

### 6.2. Algorithm: Artificial Intelligence-Driven Imaging and Recognition

#### 6.2.1. Deep Learning

Deep learning is the core technique for infrared fault identification but still requires improvements in generalization, real-time performance, and interpretability.

First, the construction and optimization of dedicated datasets form the foundation. Currently, infrared image datasets of power equipment face multiple challenges, including insufficient sample numbers, incomplete scene coverage, and limited annotation accuracy [4,138]. Large-scale, high-quality professional datasets will be constructed in the future, covering different climatic conditions such as high temperature, low temperature, rain, snow, and haze, including various equipment types such as transformers, transmission lines, switch cabinets, and insulators, and encompassing different fault types such as heating, discharge, insulation defects, and mechanical damage. The total sample quantity is expected to exceed one million pieces. At the same time, semi-supervised learning, weakly supervised learning, and generative adversarial network (GAN) technologies will be employed, effectively improving model identification capability for small samples and rare faults while reducing annotation costs. For example, generating diverse fault samples using GAN can improve the accuracy of identifying rare failures such as insulator flashover precursors by 30 percent or more [139,140].

Second, the design and deployment of lightweight networks is key. Traditional networks such as ResNet and YOLO impose excessive computational burden on resource-limited drones and inspection robots, necessitating the development of more lightweight network structures. Through model pruning, quantization compression, and knowledge distillation, model volume can be reduced to one-tenth or one-fifth of the original size, with inference speed increasing to more than 30 frames per second while maintaining recognition accuracy. For example, lightweight networks such as MobileNet and ShuffleNet enable real-time fault identification. Through structural optimization including depthwise separable convolution and channel rearrangement, latency below 30 ms can be achieved on embedded GPUs such as the Jetson Xavier NX [140,141].

Third, breakthroughs in image restoration and enhancement technology are important for improving diagnostic reliability. Infrared imaging is susceptible to noise, blur, artifacts, and uneven illumination, resulting in insufficient fault feature extraction. In the future, deep learning-based image restoration technologies require further development to improve image quality under low illumination, long-distance, and complex background conditions. For example, combining convolutional neural networks (CNNs) with transformer-based image denoising models can effectively remove Gaussian noise and salt-and-pepper noise from infrared images, improving peak signal-to-noise ratio (PSNR) by 10 to 15 dB. Super-resolution reconstruction technology can enhance low-resolution infrared image resolution by four times [139,141], clearly revealing micro-fault characteristics such as wire breakage and insulator cracks.

Finally, development in multimodal fusion diagnosis technology represents the development direction for enhancing comprehensive diagnostic capability. Single infrared imaging modes are prone to misjudgment in complex scenes. Fusing multimodal data including visible light, ultraviolet, ultrasonic, partial discharge, and insulation resistance enables construction of a multi-dimensional fault feature library, enhancing the reliability of diagnostic results. For example, an attention mechanism-based multimodal fusion network can adaptively assign weights to each modal data. Combining temperature information from infrared images, structural information from visible light images, and internal defect information from ultrasonic data can improve fault identification accuracy to 99 percent or higher, with the misjudgment rate can be reduced to 0.5 percent or less [139,140].

#### 6.2.2. Compressed Sensing Technology

Through sparse sampling and reconstruction algorithms, compressed sensing technology can accurately reconstruct signals at sampling rates far below the Nyquist rate. In the future, compressed sensing applications in autonomous power inspection infrared imaging will focus on three directions.

The first is scene-adaptive sparse sampling matrix design. Targeting power patrol inspection imaging scenes, the sparse characteristics of infrared images are considered, including local concentration of fault areas and sparse distribution of edge features. An adaptive sampling matrix is designed to reduce the sampling rate to 10 to 30 percent, reducing data transmission volume and storage requirements while ensuring image quality. For example, a structured random sampling matrix is designed based on statistical characteristics of power equipment infrared images [142].

The second is fast reconstruction algorithm optimization. Traditional compressed sensing reconstruction algorithms such as orthogonal matching pursuit and basis pursuit have high computational complexity, making them difficult to satisfy real-time imaging requirements. In the future, deep learning-based fast reconstruction algorithms should be developed to leverage the powerful fitting capability of neural networks to achieve real-time infrared image reconstruction. For example, a CNN-based compressed sensing reconstruction network can achieve reconstruction speeds exceeding 100 frames per second on GPUs. Reconstruction error is reduced to less than five percent [143,144], satisfying real-time requirements for dynamic patrol inspection.

Third is the cooperative design of detectors and compressed sensing. By integrating compressed sensing technology into detector array design, novel compressed sensing-based detector readout circuits are developed, reducing the number of pixels in the array, lowering detector cost and power consumption. For example, a reconfigurable detector array based on micro-electro-mechanical systems (MEMSs) randomly selects a subset of pixels for sampling. Combined with a compressed sensing reconstruction algorithm, this reduces pixel count by 50 percent while maintaining imaging quality, with detector cost reduced by 30 to 40 percent [142,143].

#### 6.2.3. Cross-Modal Fusion Strategy

Cross-modal fusion integrates complementary detection modalities and has become a core direction for autonomous power inspection, enabling comprehensive and precise fault diagnosis. Future developments will focus on three main areas: constructing multimodal data association models, optimizing unified representation and fusion algorithms, and advancing fault prediction and early warning technologies. In terms of constructing multimodal data association models, it is necessary to establish data association between infrared imaging and other detection modalities, including partial discharge ultrasonic detection, insulation resistance testing, and equipment operational data. This allows in-depth exploration of intrinsic relationships between different modalities, achieving complementarity and validation of fault features. For example, by building an association model between infrared temperature and partial discharge intensity, when infrared detects a local device temperature exceeding 80 °C and ultrasonic detection detects partial discharge signals [145,146], it can be determined as a serious fault, triggering the emergency maintenance process. In terms of unified representation and fusion algorithm optimization, a unified representation method for cross-modal data must be developed to convert detection results from different modalities into unified feature vectors. Using feature-level and decision-level fusion techniques, a comprehensive quantitative evaluation of power equipment health status can be completed. For example, autoencoders can achieve unified representation of infrared images, ultrasonic signals, and operational data. After extracting multimodal shared features, decision-level fusion algorithms such as weighted voting and Bayesian inference can output a 0 to 100 range equipment health index [145], enabling quantitative assessment of faults. In terms of breakthroughs in fault prediction and early warning technologies, it is necessary to combine cross-modal data with historical equipment fault records. Techniques such as deep learning and time series analysis build fault prediction models to enable early warning of potential faults. For example, a LSTM network can predict fault risks 1–3 months in advance by taking as input the equipment’s infrared temperature, partial discharge, and load data. Early warning accuracy exceeds 85 percent. This promotes the shift of power inspections from post-event diagnosis to pre-event prediction, providing more comprehensive and proactive technical support for the safe and stable operation of power systems [147].

#### 6.2.4. Other Intelligent Algorithms

In addition to the core computational methods mentioned above, other intelligent algorithms will also be widely applied in infrared imaging for autonomous power inspection, including reinforcement learning, transfer learning, and federated learning.

Reinforcement learning can optimize patrol paths and imaging parameters. By constructing a reward mechanism for the patrol environment (e.g., fault detection rate, patrol efficiency, and energy consumption), a reinforcement learning agent is trained, enabling patrol equipment to adaptively adjust shooting angle, focal length, exposure time, and patrol path based on environmental changes and equipment status. This improves patrol efficiency and imaging quality. For example, a UAV patrol path optimization algorithm based on deep reinforcement learning can plan optimal patrol paths in complex terrain environments, reducing flight time by more than 30 percent while ensuring full equipment coverage.

Transfer learning addresses the problem of fault feature migration between different power equipment types. Different equipment (e.g., transformers, transmission lines, and switch cabinets) exhibit distinct fault characteristics. Traditional models require separate training for each equipment type, resulting in high costs. Transfer learning allows trained model parameters to be transferred to fault identification tasks for new equipment, reducing the number of labeled samples needed and lowers training costs. For example, migrating a model trained on transformer fault data to switchgear fault identification requires only a small number of switchgear samples for fine-tuning, achieving accuracy exceeding 95 percent.

Federated learning enables joint training of inspection data across multiple regions and facilities. Under the premise of protecting data privacy, multiple power enterprises can collaboratively train fault identification models, enhancing model generalization ability. For example, adopting a federated learning distributed training framework allows each enterprise to train models locally. Only model parameter updates are shared, while original data is not transmitted, protecting data privacy while improving fault identification accuracy across different regions by 5 to 10 percent.

### 6.3. System: Integration and Cooperative Operation

#### 6.3.1. Inspection and Upgrade of Autonomous Robots

Autonomous robots are the primary platforms for infrared imaging inspection. Future development focuses on multi-platform coordination, high-precision navigation, long endurance, and environmental robustness, building a patrol inspection system covering all scenarios.

Integrated UAVs, ground robots, wall-climbing robots, and autonomous patrol vehicles form a multi-dimensional cooperative patrol network, creating an air–ground–wall inspection framework. UAVs are responsible for inspecting transmission lines, tower tops, and other high-altitude scenarios. Ground robots handle indoor and outdoor flat scenes such as substation yards and distribution rooms. Wall-climbing robots perform close-range inspections of large equipment enclosures including transformers and reactors. Autonomous patrol vehicles support ground-based auxiliary patrols and data relay for long-distance transmission lines. Through multi-platform coordination, power equipment can be monitored without blind spots, increasing patrol coverage to 100 percent [148,149].

Navigation technologies including LiDAR, vision SLAM, GPS, or BeiDou positioning, and inertial navigation are integrated to enhance robot positioning accuracy and obstacle avoidance capability in complex environments. For example, a fusion navigation scheme combining LiDAR and visual SLAM achieves centimeter-level positioning accuracy of ±5 cm in indoor substation scenarios without GPS signals. A deep learning-based obstacle avoidance algorithm identifies dynamic targets such as branches, birds, and other obstacles. Obstacle avoidance response time remains below 0.5 s [121,150], ensuring patrol safety.

Optimization of robot mechanical structures and power systems improves load capacity. Miniaturized, low-power infrared imaging systems, data processing modules, and communication modules are integrated. For example, UAV payload capacity increases to 5 to 10 kg, enabling simultaneous deployment of infrared cameras, visible light cameras, and partial discharge detectors. High-energy-density batteries, supplemented by solar-assisted charging, extend UAV endurance to 4–8 h. Ground robot endurance extends to 12 to 24 h [148,149], meeting the requirements for large-scale and long-duration patrol inspections.

Multi-robot cluster algorithms support task allocation, data sharing, and cooperative inspection. For example, based on distributed consensus algorithms, multiple UAVs automatically allocate patrol areas, avoiding redundant patrols and omissions. Through 5G/6G communication technology, real-time data sharing among robots is achieved. When one robot detects a fault, it can dispatch other robots for multi-angle review and detailed inspection, improving fault location accuracy [149].

#### 6.3.2. Edge Computing Deployment

Edge computing processes data locally, reducing latency and improving real-time decision-making. It has become indispensable key technological support for autonomous power inspection infrared imaging systems. Future deployment optimization will focus on three core areas, including the construction of dedicated edge computing nodes, the development of adaptive computing power scheduling algorithms, and the optimization of edge storage and data management.

In terms of dedicated edge computing node construction, research will closely align with specific scenario requirements of power inspections. Compact, low-power, and highly reliable edge computing nodes will be developed. By integrating heterogeneous computing units such as GPUs and FPGAs, these nodes fully meet diverse computing demands, including deep learning model inference, image processing, and data storage. For example, FPGA-based edge computing nodes maintain power consumption within 5 to 10 watts. They efficiently perform real-time fault detection, image compression, and data encryption. Latency is controlled within 50 ms, meeting the stringent requirements of power inspections for device portability, battery life, and real-time performance [151].

In developing adaptive computing power scheduling algorithms, computing resources will be dynamically and intelligently allocated. This allocation is based on the complexity of inspection tasks such as fault detection, image reconstruction, and multimodal fusion. It also considers real-time device status including remaining battery power and network bandwidth. The goal is to achieve an optimal balance between computing performance and power consumption. For example, when the device has sufficient battery power and ample network bandwidth, a high-precision model will be used for fault detection, ensuring the accuracy of diagnostic results. When battery power is low or network bandwidth is limited, the system automatically switches to a lightweight model, maintaining essential functionality while conserving resources [152]. Priority is given to ensuring the stable operation of core inspection functions, avoiding task interruptions caused by insufficient resources. In terms of optimizing edge storage and data management, an integrated storage architecture will be constructed. Edge storage and cloud storage work collaboratively in this architecture. Edge nodes primarily handle local data caching, preprocessing, and incremental upload tasks. The cloud is responsible for long-term data storage and global analysis. For example, edge nodes will selectively store important information such as fault images and key inspection data. Data is compressed to 10 to 20 percent of its original volume. Incremental data is then uploaded to the cloud via 5G-Advanced or 6G networks, greatly reducing network bandwidth usage. At the same time, local data encryption technologies and strict access control techniques will be employed on edge computing nodes, comprehensively ensuring the security and integrity of inspection data and preventing risks such as data leakage or tampering during storage and transmission. This provides solid data support for the stable and efficient operation of autonomous power inspection infrared imaging systems.

#### 6.3.3. Cloud Edge Collaboration Architecture Construction

Cloud edge collaboration combines edge real-time processing with cloud-scale computing and storage. It constructs an integrated system of perception, edge processing, and cloud decision, which represents the core architecture for future autonomous power inspection infrared imaging systems.

A power patrol cloud platform is built to enable centralized storage, management, analysis, and visualization of massive patrol data. The cloud platform has three core functions. The data management function supports unified storage, indexing, and querying of multi-source data, including infrared images, visible light images, and equipment operational data. The data analysis function leverages cloud computing power for model training, fault trend analysis, and equipment health status evaluation. The visualization function uses GIS mapping and 3D modeling technologies to visually display patrol paths, fault locations, and equipment health status. This supports remote monitoring and decision-making [153,154].

Efficient and reliable cloud edge cooperative data transmission protocols are designed, supporting real-time data synchronization, instruction interaction, and breakpoint resumption. For example, protocols such as MQTT and HTTP/2 enable low-latency communication between edge nodes and the cloud. Data transmission delay remains below 100 ms [155]. Standardized data interfaces and command interfaces are established, achieving interconnection and interoperability between inspection equipment from different manufacturers and the cloud platform [153].

An intelligent decision-making system is developed that collaborates across cloud, edge, and terminal layers. It combines global data from the cloud with real-time data from the edge, enabling hierarchical fault warnings, automatic work order generation, maintenance resource scheduling, and maintenance effectiveness evaluation. For example, when an edge node detects a serious fault, the cloud platform automatically generates a maintenance work order. It sends this order to the relevant maintenance personnels mobile application. It optimizes maintenance resource scheduling based on personnel location and skill levels. After maintenance is completed, inspection robots are dispatched for re-inspection. This forms a closed-loop management system of detection, warning, maintenance, and re-inspection [153,154], which significantly improves inspection and maintenance efficiency.

#### 6.3.4. Other Systems

In addition to the core directions mentioned above, other technologies will also become important components of system optimization, including dynamic range expansion, wireless communication integration, and self-calibration with self-diagnosis capabilities for infrared imaging systems.

Dynamic range expansion technology will address imaging challenges in high-dynamic-range scenarios. Infrared imaging of power equipment often faces scenes where high-temperature conductors and low-temperature backgrounds coexist, with dynamic ranges exceeding 100 dB. In the future, multi-exposure fusion, HDR imaging, and adaptive gain adjustment will be adopted. These technologies will expand the dynamic range of infrared imaging systems to over 120 dB, enabling clear imaging of both high-temperature conductors and low-temperature backgrounds simultaneously.

Wireless communication integration technology will improve data transmission speed and reliability. By integrating 5G-Advanced/6G, WiFi 7, LoRa, and other wireless communication technologies, a communication network with high data rate, low latency, and wide coverage will be established. 5G technology supports high-speed transmission of high-definition images and video data, achieving transmission rates above 10 Gbps. WiFi 6 enables high-speed communication in short-range scenarios such as indoor substation environments. LoRa provides long-distance, low-power data transmission with communication distances exceeding 10 km. Through the integration of multiple communication technologies, real-time and reliable transmission of patrol inspection data is ensured.

Self-calibration and self-diagnosis technologies will enhance system reliability and maintenance convenience. Self-calibration algorithms for infrared detectors will be developed. Using built-in standard blackbody sources and temperature sensors, automatic calibration of parameters such as detectivity and responsivity can be achieved, with calibration accuracy reaches ±1 percent. A system self-diagnosis model will be constructed to monitor the working status of key components including detectors, lenses, communication modules, and power modules. This enables early warning of faults and potential self-repair capabilities. Maintenance costs and system downtime are consequently reduced.

### 6.4. Standardization and Industrialization Path: Specification and Implementation Promotion

#### 6.4.1. Construction of Standardization System

Standardization is the core prerequisite for large-scale adoption of infrared imaging technology in autonomous power inspection. A comprehensive, full-chain standardization system will be established, laying the foundation for technical implementation and industrial coordination.

A forward-looking standardization framework must be grounded in a clear understanding of technology iteration cycles. Currently, infrared data transmission has widely adopted USB 3.1 Gen 1 (5 Gbps) and above, while edge computing FPGAs have entered the 7 nm node and beyond (e.g., AMD Versal AI Edge series), delivering an order-of-magnitude improvement in AI engine throughput and energy efficiency compared with 28 nm platforms. Therefore, the proposed standardization system mandates that core interface protocols, computing platform selections, and communication standards be benchmarked against mainstream commercial solutions at the time of standard release, with a mandatory biennial review and update mechanism. This approach prevents the standardization process from locking in outdated technologies and ensures that the entire design chain—from materials and devices to systems—is not constrained by obsolete assumptions, thereby establishing a self-correcting and continuously modernizing technical foundation.

In terms of equipment technical standard formulation, a core technical indicator system for infrared imaging patrol inspection equipment will be defined [156,157]. Detector performance must meet specific requirements. Detection rate D* shall be no less than 10^10^ Jones. Response time shall be no more than 10 ms. Spectral response range shall cover 3 to 12 micrometers. Imaging quality standards require resolution of at least 640 by 512 pixels. Signal-to-noise ratio shall be no less than 50 dB. Image uniformity shall be within five percent. Environmental adaptability specifications include an operating temperature range of −40 °C to 85 °C. Protection level shall be at least IP65. Anti-electromagnetic interference capability shall withstand 200 volts per meter. Equipment mean time between failures shall exceed 10,000 h to ensure reliability. Whole-process standards for design, production, testing, and calibration will regulate market access and ensure consistent product quality.

In terms of establishing fault diagnosis standard specifications, classification rules, diagnosis thresholds, and evaluation methods for infrared faults of power equipment will be unified. Normal temperature ranges, abnormal temperature warning thresholds, and critical fault temperature criteria will be defined for different equipment types including transformers, transmission lines, and insulators. Four fault severity levels will be established: minor fault, general fault, serious fault, and emergency fault. Corresponding treatment procedures and maintenance time limits will be specified for each level. A fault diagnosis traceability mechanism will be established. Complete recording of detection data, diagnosis processes, and decision-making basis will ensure verifiability and traceability of diagnostic results.

In terms of data format and interface specification unification, standard formats for patrol inspection data will be developed, covering infrared images, visible light images, fault description information, equipment parameters, and other content. General data formats such as JSON and XML will be adopted [157], breaking down data barriers between different devices and platforms. Protocols and standards for data transmission interfaces, control command interfaces, and calibration interfaces between devices and cloud platforms, as well as among devices themselves, will be unified. This enables cross-manufacturer device compatibility, cross-regional inspection collaboration, and data exchange and sharing [156]. Interface specifications will mandate support for current-generation high-speed protocols (e.g., USB 3.2/4.0, PCIe 4.0/5.0) and define forward-compatible migration paths to emerging standards.

To strengthen the industry certification framework, a third-party institution dedicated to equipment performance testing and calibration will be established. Rigorous certification protocols and technically robust standards will be developed and implemented. All infrared imaging detection equipment will undergo comprehensive performance evaluation and regulatory compliance verification, followed by the issuance of authoritative certification documents. Certification serves as a gatekeeping mechanism: nonconforming or substandard products will be systematically excluded from the market, thereby incentivizing enterprises to prioritize technological developments and continuous quality enhancement. Furthermore, a standardized equipment calibration and annual inspection regime will be institutionalized, with clearly specified calibration intervals, inspection scopes, and pass or fail criteria. This structured oversight ensures sustained equipment reliability and operational stability, facilitating the large-scale deployment of domestically developed infrared imaging technology in power system inspection applications [158].

#### 6.4.2. Key Technology Breakthrough of Industrialization

Industrialization constitutes a critical enabler for the practical deployment and widespread adoption of infrared imaging technology in autonomous power inspection. Future efforts will prioritize four strategic domains: (1) scaling up detector manufacturing to meet growing demand; (2) integrating end-to-end inspection systems (including hardware, software, and operational workflows); (3) establishing robust, standardized quality assurance frameworks; and (4) strengthening collaborative innovation ecosystems among industry, academia, and research institutions.

In the domain of large-scale manufacturing technologies for infrared detector arrays, strategic emphasis will be placed on optimizing critical process steps—including photolithography, thin-film deposition, and wafer-level packaging of uncooled infrared detector arrays. These process enhancements are expected to significantly improve production throughput while concurrently reducing unit manufacturing costs. Notably, the adoption of an 8-inch wafer-level fabrication platform enables high-volume, uniform production; annual output capacity exceeds 100,000 detector array units, and per-unit manufacturing cost is reduced by 50–67% relative to prior-generation processes. Concurrently, advanced fabrication methodologies will be developed to achieve exceptional inter-pixel consistency across large-format arrays. Target pixel uniformity—quantified as response consistency across the array—is projected to exceed 98%, thereby satisfying stringent performance requirements for large-scale thermal imaging systems.

With regard to integrated detection equipment, a multi-module integration architecture will be pursued, incorporating infrared imaging, inertial navigation, real-time data processing, broadband communication (e.g., 5G), and intelligent power management subsystems. R&D efforts will prioritize miniaturization, weight reduction, and cost efficiency without compromising functional integrity or operational reliability. As a representative implementation, a compact integrated detection unit—integrating a high-sensitivity infrared camera, an edge AI computing module, and a low-latency 5G communication interface—has been designed. Its dimensions are constrained to 10 cm × 10 cm × 5 cm, total mass is under 1 kg, and overall system cost is reduced by more than 30% compared with legacy discrete-system configurations. A standardized modular design paradigm will be adopted to support streamlined field maintenance, rapid hardware–software co-upgrades, and scalable system reconfiguration. Finally, a comprehensive, end-to-end quality management system will be implemented—spanning raw material procurement, component fabrication, subsystem assembly, system integration, and final delivery. This framework ensures full traceability of product quality throughout the lifecycle. Multiple in-process inspection checkpoints will be institutionalized, key process parameters (e.g., film thickness, alignment accuracy, thermal budget) will be monitored in real time via automated metrology, and dynamic process corrections will be enabled. A centralized digital quality repository will systematically archive device-specific production logs, environmental test records, electro-optical calibration data, and final acceptance reports—thereby guaranteeing auditable, granular traceability for every delivered unit [156].

In terms of advancing the industry–university–research collaboration model, deeper integration among universities, research institutions, and enterprises will be actively promoted. A comprehensive development chain encompassing basic research, technology development, product transformation, and market application will be established. Enterprises will assume a leading role in the industrialization and translation of technological achievements. They will respond to market demands with precision to drive the development of practical products. Government entities will contribute policy guidance and financial support, facilitating the construction of an effective cooperation platform. Such efforts will accelerate the application and commercial deployment of technological promotions [158].

#### 6.4.3. Commercialization Promotion Strategy

Commercialized promotion constitutes the key mechanism for realizing the market value of infrared imaging technology in autonomous power inspection.

Customized inspection products and solutions will be developed to address the distinct operational requirements of diverse user groups, including the State Grid, China Southern Power Grid, regional power companies, and power operation and maintenance enterprises. For instance, an integrated air–ground–wall multi-platform collaborative inspection solution will be provided to large-scale power utilities such as the State Grid, tailored to meet the demands of large-area and cross-regional inspection tasks. Local power companies will be equipped with low-cost, miniaturized inspection devices suitable for small- to medium-scale operations. Portable, high-performance infrared imaging systems will be supplied to power operation and maintenance enterprises to facilitate on-site diagnostic and inspection activities.

In parallel with equipment sales, a portfolio of value-added services will be expanded, encompassing cloud-based storage of inspection data, fault diagnosis and analysis, equipment health management, and technical training. This strategy establishes a diversified revenue model integrating equipment sales with value-added services. For example, a cloud storage service for inspection data will be offered, with pricing structured according to storage capacity and service duration. Professional fault diagnosis and analysis services will deliver comprehensive diagnostic reports and maintenance recommendations. Technical training programs will equip users with specialized skills in equipment operation, fault identification, and routine maintenance.

Demonstration application projects will be implemented in critical power infrastructure and complex inspection environments. These initiatives serve to validate product performance and reliability while building a portfolio of application cases, thereby enhancing market acceptance. For instance, inspection efficiency and fault identification accuracy under complex environmental conditions will be validated through deployments on UHV transmission lines, large-scale substations, and transmission lines traversing challenging terrain. Successful demonstration outcomes will underpin market promotion strategies and attract broader adoption.

International technical collaboration and knowledge exchange with leading foreign enterprises and research institutions will be actively pursued. Advanced foreign technologies and expertise will be assimilated to elevate the technical standards of domestic products. Concurrently, the global promotion of domestic inspection equipment will be advanced through participation in international markets. Engagement in global power equipment exhibitions will showcase the technological advantages of domestically developed inspection systems. Strategic partnerships with foreign power utilities will be cultivated to implement overseas demonstration projects and expand into international markets.

#### 6.4.4. Policy and Ecological Support

Policy and ecological support constitute a critical foundation for advancing the industrialization of infrared imaging technology in autonomous power inspection. Moving forward, a conducive environment for technological progress will be cultivated through strategic policy initiatives, the development of an integrated industrial ecosystem, and systematic talent cultivation.

Efforts will be directed toward integrating infrared imaging intelligent inspection technology into national science and technology strategies and industrial support frameworks within the power sector. Enhanced investment in research and development, alongside market promotion initiatives, will be actively pursued. For instance, dedicated research and development funding programs will be established to support advanced development in novel materials, core algorithms, and fundamental technologies. Incentive policies, including subsidies, will be introduced to encourage power enterprises to adopt domestically produced inspection equipment. Furthermore, infrared imaging intelligent inspection technology will be incorporated into industry-wide standard specifications and technology promotion catalogs, thereby facilitating its large-scale deployment and application.

Concurrently, the active participation of upstream and downstream enterprises will be encouraged to foster collaborative industrial development. This includes material suppliers, device manufacturers, software developers, and operation and maintenance service providers. Such engagement will contribute to the formation of synergistic industrial clusters. For example, specialized industrial parks will be established to concentrate enterprises across the value chain, enabling resource sharing and the realization of complementary advantages. Industry platforms will be developed to facilitate technical collaboration and knowledge exchange among enterprises, thereby promoting synergistic development throughout the industrial ecosystem. A cohort of leading enterprises with core technological competencies will be cultivated to drive the advancement of the entire industry.

Interdisciplinary talent development will be prioritized to cultivate professionals possessing integrated expertise in electric power systems, infrared imaging technology, artificial intelligence algorithms, and robotics. This strategic focus on human capital development will provide essential intellectual support for sustained industrial growth [156]. For instance, academic institutions will establish specialized curricula to cultivate interdisciplinary expertise at the undergraduate, masters, and doctoral levels. University–industry partnership programs will be implemented to strengthen practical training and enhance students applied competencies. Sector-specific training frameworks and professional certification mechanisms will be developed to advance the technical proficiency of incumbent practitioners. Additionally, internationally renowned experts will be recruited to mitigate domestic talent deficiencies and accelerate technological advancement.

**Figure 11:**
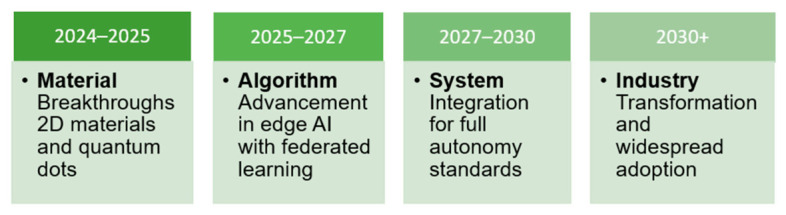
Roadmap for the future development of intelligent infrared inspection technology. This timeline outlines the key stages of technological evolution in intelligent infrared inspection, from material breakthroughs to full industry transformation, spanning from 2024 to beyond 2030.

In conclusion, the future trajectory of infrared imaging technology for autonomous power inspection follows a clearly defined evolutionary pathway. The corresponding technology roadmap is presented in Figure 11, which systematically delineates the complete development chain from fundamental material discoveries to large-scale industrial deployment. This roadmap provides strategic guidance for subsequent research directions and informs the formulation of long-term industrial planning.

## 7. Summary

In summary, this review has examined the technological landscape of infrared imaging for autonomous power inspection, spanning from detector innovation to system integration. Motivated by the limitations of manual inspection—namely low efficiency, high subjectivity, and poor accessibility—the transition toward intelligent, data-driven maintenance has become imperative in the context of smart grids and Industry 4.0. Infrared imaging, with its non-contact, visual, and predictive capabilities, stands as a cornerstone technology for this transformation.

The key conclusions drawn from this review are threefold. First, significant progress has been made in infrared detector technology: uncooled microbolometers have enabled cost-effective and portable solutions, while emerging low-dimensional materials—such as two-dimensional materials and quantum dots—offer disruptive potential by narrowing the performance gap between sensitivity, operability, and cost. Second, the integration of advanced signal processing (e.g., real-time non-uniformity correction, adaptive noise suppression) and innovative optical systems (e.g., computational imaging, lensless designs) has proven essential for enhancing image quality and diagnostic reliability in complex field environments. Third, the convergence of these technologies with autonomous platforms—drones, ground robots, and edge computing—has resulted in measurable improvements in inspection efficiency, fault detection accuracy, and economic benefits across transmission lines, substations, and photovoltaic plants.

Despite these advances, several limitations remain. Novel detectors still face challenges in long-term stability, large-scale manufacturability, and environmental robustness. End-to-end optimized systems impose high computational demands that are not yet fully resolved in resource-constrained mobile platforms. Furthermore, the lack of standardized protocols for data formats, device interfaces, and fault diagnosis continues to hinder interoperability and widespread adoption.

In perspective, while infrared imaging has matured considerably for autonomous power inspection, its full potential hinges on overcoming material stability issues, reducing system complexity, and establishing industry-wide standards. Future efforts should prioritize high-performance uncooled arrays, AI-driven optical algorithm co-design, and the development of standardized, low-cost, and intelligent inspection platforms. Addressing these challenges will be critical for realizing fully autonomous, reliable, and scalable power infrastructure maintenance. Looking ahead, infrared imaging for autonomous power inspection is poised to gradually transform from semi-autonomous assistance to fully autonomous closed-loop operation. With continued advances in materials science and integration technologies, infrared detectors are set to achieve performance enhancement, cost optimization, and dimensional miniaturization. Intelligent algorithms will exhibit stronger adaptability to complex inspection conditions. System integration will evolve toward higher integration, synergistic cooperation, and intelligent upgrading. Such advances may facilitate the establishment of standardized product systems featuring high integration, low cost, and modular design, broadening the industrial application scope of the proposed technology, further providing robust technical support for the power industry’s transition toward digital, intelligent, and unmanned modern operation and maintenance systems. In the foreseeable future, infrared imaging is expected to play an increasingly important role in supporting the safe and stable operation of critical power infrastructure.

## Figures and Tables

**Table 1:** Comparison of typical infrared detector performance and trade-off analysis in power inspection scenarios.

Detector Type	NETD (°C)	Cost (k RMB)	Response Band (μm)	Applicable Power Inspection Scenarios
HgCdTe (Cooled) (Refs. [67,68])	0.01	80	3–5	Deep fault diagnosis (transformers, GIS)
InSb (Cooled) (Ref. [69])	0.015	50	1–5	Cable joints, substation short-range
QWIP (Cooled) (Ref. [70])	0.02	40	3–12	Large-area scanning (insulators, substations)
Uncooled microbolometer (VOx/a-Si) (Ref. [71])	0.03	8	8–14	Routine patrol (UAVs/robots, PV hot spots)
Black phosphorus (2D) (Ref. [72])	0.025	20	3–9	Small UAVs, multi-band conformal inspection
Quantum dot (PbS/PbSe) (Ref. [73])	0.04	6	1–5	Distributed/portable terminals

**Table 2:** Comparison of different signal processing algorithm performances.

Algorithm Type	Core Function	Platform	Latency	Effect	Application
Two-point calibration (Ref. [74])	Non-uniformity correction	FPGA	≤0.1 ms	Error ± 2%	Basic calibration
Five-point calibration (Ref. [75])	Nonlinear correction	FPGA + AI chip	≤20 ms	Error ≤ ±0.5%	High-precision temp. measurement
Scene-based gradient algorithm (Ref. [76])	Pot lid effect + non-uniformity correction	FPGA	0.6 ms	Effective area ≥ 99%	UAV/robot inspection
Inter-frame + weighted guided filter (Ref. [77])	Random noise suppression	FPGA	<1 ms	BIQI ↑ 4.80–9.31	Outdoor complex environments
Improved YOLOv5 (CBAM + EIoU) (Ref. [78])	Fault target detection	AI chip (Ascend 310)	≤30 ms	mAP = 93.81%, FPS = 145.64	PV/transmission line fault detection

Note: The upward arrow (↑) indicates that a higher BIQI value corresponds to better image quality.

**Table 3:** Comparison of image quality (PSNR/SSIM) of different algorithms in power inspection scenarios.

Algorithm Type	Test Scenario	PSNR (dB)	SSIM	Core Advantages	Application
Traditional median filtering + two-point calibration (Ref. [101])	Normal sunny day	28.6 ± 1.2	0.79 ± 0.03	Low complexity, high real-time	Short-distance routine inspection (distribution rooms)
Gaussian filtering + multi-point calibration (Ref. [102])	Strong EMI (substation)	26.3 ± 1.5	0.75 ± 0.04	High EMI resistance	Short-distance temp. measurement (substation)
Improved inter-frame + weighted guided filtering (Ref. [103])	Foggy day (Visibility < 50 m)	32.8 ± 0.9	0.88 ± 0.02	Excellent scattering suppression	UAV inspection in mountainous/foggy areas
Non-local means filtering (Ref. [104])	Long-distance (≥500 m)	30.5 ± 1.1	0.83 ± 0.03	Good detail & blur suppression	Long-distance fault identification (HV lines)
CNN-based image enhancement (Ref. [105])	Complex mixed environment (Fog + EMI)	35.2 ± 0.8	0.92 ± 0.01	Strong multi-interference adaptation	Complex terrain substation/line inspection

**Table 4:** Technical parameters and performance comparison of typical scenarios for power inspection.

Application Scenario	Platform	Core Configuration	Key Parameters	Efficiency	Detection Accuracy	Adaptability	Economic Benefits
UAV transmission line inspection (Ref. [106])	Multirotor UAV	640 × 512 thermal imager, RTK-GNSS, 3D LiDAR	Endurance ≥ 60 min, GSD 1.7 cm/pixel, accuracy ±3 cm, thermal sensitivity ≤ 0.05 °C	5 km/h, 10× faster than manual	Joints: 98.2%, insulators: 97.5%	All-weather; fog (<50 m), wind (≤15 m/s)	80% cost reduction; avoids > 10 M RMB losses
Substation inspection robot (Ref. [107])	Ground wheeled robot	640 × 512 thermal imager, 3D laser navigation, liftable PTZ	Accuracy ±2 cm, IP67, sensitivity 0.05 °C, speed 0.5 m/s	Whole substation in 60 min, 3× faster than manual	Transformers: 98.7%, GIS: 97.6%, switches: 98.2%	High EMI resistance (>1000 V/m), rain, snow, sand	Outage time reduced from 24 h → 3 h; reliability +87.5%
PV plant fault diagnosis (Ref. [108])	Multirotor UAV	640 × 512 thermal imager, GPS, LiDAR	Endurance ≥ 60 min, GSD 1.7 cm/pixel, 15 fps	10 MW plant in 45 min, 10–15× faster than manual	Severe hot spots: 99.1%, diode failure: 94.7%, junction box: 98.3%	High temp (40 °C), fog (<50 m)	Generation efficiency +3.2%; outage loss—83.3%

Note: The arrow (⟶) indicates the change of the parameter value before and after optimization.

## Data Availability

No new data were created.

## References

[1] Huang J., Xu J., Meng L., Qin Z. (2016). The Comprehensive Benefit Evaluation Model of Manual Inspection in Transmission Line. Proceedings of the 6th International Conference on Electronic, Mechanical, Information and Management Society, Shenyang, China, 1–3 April 2016.

[2] Jaffery Z.A., Dubey A.K., Haque A. (2017). Scheme for predictive fault diagnosis in photo-voltaic modules using thermal imaging. Infrared Phys. Technol..

[3] Slaughter D.C., Crisosto C.H., Hasey J.K., Thompson J.F. (2006). Comparison of Instrumental and Manual Inspection of Clingstone Peaches. Appl. Eng. Agric..

[4] Chen J., Liu Y., Li S., Lin L., Li Y., Huang W., Guo J. (2024). Ferroelectric-controlled graphene plasmonic surfaces for all-optical neuromorphic vision. Sci. China Technol. Sci..

[5] Guo J., Gu S., Lin L., Liu Y., Cai J., Cai H., Tian Y., Zhang Y., Zhang Q., Liu Z. (2024). Type-printable photodetector arrays for multichannel meta-infrared imaging. Nat. Commun..

[6] Ha S.T., Li Q., Yang J.K.W., Demir H.V., Brongersma M.L., Kuznetsov A.I. (2024). Optoelectronic metadevices. Science.

[7] Liu F., Zheng H., Ma S., Zhang W., Liu X., Chua Y., Shi L., Zhao R. (2024). Advancing brain-inspired computing with hybrid neural networks. Natl. Sci. Rev..

[8] Jadidi R., Badihi H., Zhang Y. (2020). Fault Diagnosis in Microgrids with Integration of Solar Photovoltaic Systems: A Review. IFAC Pap..

[9] Korki M., Shankar N.D., Shah R.N., Waseem S.M., Hodges S. (2019). Automatic Fault Detection of Power Lines using Unmanned Aerial Vehicle (UAV). 2019 1st International Conference on Unmanned Vehicle Systems-Oman (UVS).

[10] Xu C., Li Q., Zhou Q., Zhang S., Yu D., Ma Y. (2022). Power Line-Guided Automatic Electric Transmission Line Inspection System. IEEE Trans. Instrum. Meas..

[11] Constantin A., Dinculescu R.N. UAV development and impact in the power system. Proceedings of the 2019 8th International Conference on Modern Power Systems (MPS).

[12] Nguyen V.N., Jenssen R., Roverso D. (2018). Automatic autonomous vision-based power line inspection: A review of current status and the potential role of deep learning. Int. J. Electr. Power Energy Syst..

[13] Hütten N., Alves Gomes M., Hölken F., Andricevic K., Meyes R., Meisen T. (2024). Deep Learning for Automated Visual Inspection in Manufacturing and Maintenance: A Survey of Open-Access Papers. Appl. Syst. Innov..

[14] da Silva M.F., dos Santos M.F., Masson J.E.N., Alves P.M.R., Silva W.R., Martins G.M.C., da Costa V.H.G., Schettino V.B., Stivanello M.E. (2025). AI-Powered Automated Inspection for Optimized Asset Management in Electrical Distribution Networks. J. Control Autom. Electr. Syst..

[15] Chen J., Li Y., Cao L. (2021). Research on region selection super resolution restoration algorithm based on infrared micro-scanning optical imaging model. Sci. Rep..

[16] Asundi A.K., Huang X., Xie Y., Zeng J., Yang J., Wu H. The infrared image closely spaced objects super resolution method based on sparse reconstruction under the noise environment. Proceedings of the International Conference on Optical and Photonics Engineering (icOPEN 2016).

[17] Farsaei A., Wang Y., Molavi R., Jayatilleka H., Caverley M., Beikahmadi M., Masnadi Shirazi A.H., Jaeger N., Chrostowski L., Mirabbasi S. (2016). A review of wireless-photonic systems: Design methodologies and topologies, constraints, challenges, and innovations in electronics and photonics. Opt. Commun..

[18] Machuca G., Torres S.N., Jara A., Viafora L.A., Gutiérrez P.A. Restoration and Digital Super-Resolution for Infrared Microscopy Imaging. Proceedings of the 2nd International Conference on Vision, Image and Signal Processing.

[19] Sarawade A.A., Charniya N.N. Infrared Thermography and its Applications: A Review. Proceedings of the International Conference on Communication and Electronics Systems (ICCES 2018).

[20] Solend T.A., Rødningsby A., Moen J. (2025). Impacts of infrared thermographic image blurring on UAV inspection efficiency of solar power plants. Sol. Energy.

[21] Rogalski A., Chrzanowski K. (2014). Infrared Devices and Techniques (Revision). Metrol. Meas. Syst..

[22] Yassin M.A.M., Shrestha A., Rabie S. (2023). Digital twin in power system research and development: Principle, scope, and challenges. Energy Rev..

[23] Chang B., Zhang W., Shi Y., Hu R., Zeng Y., Yan M., Guo H. Application status and development trend of infrared imaging system. Proceedings of the International Symposium on Photoelectronic Detection and Imaging 2013: Low-Light-Level Technology and Applications.

[24] Huckridge D.A., Fan J., Yang J., Ebert R. (2013). Trends in infrared imaging detecting technology. Electro-Optical and Infrared Systems: Technology and Applications X.

[25] Mayerich D., van Dijk T., Walsh M.J., Schulmerich M.V., Carney P.S., Bhargava R. (2014). On the importance of image formation optics in the design of infrared spectroscopic imaging systems. Analyst.

[26] Guo J., Li S., Lin L., Cai J., Chen J., Wang S., Gou X., Ye J., Luo Z., Huang W. (2022). Enhanced-performance self-powered photodetector based on multi-layer MoS_2_ sandwiched between two asymmetric graphene contacts. Sci. China Technol. Sci..

[27] Wu Z., Liu Y., Guo J., Huang W. (2021). A dual-band photodetector induced by hybrid surface plasmon resonance. Jpn. J. Appl. Phys..

[28] Argirusis N., Achilleos A., Alizadeh N., Argirusis C., Sourkouni G. (2025). IR Sensors, Related Materials, and Applications. Sensors.

[29] Liu X., Zhang Z., Hao Y., Zhao H., Yang Y. (2024). Optimized OTSU Segmentation Algorithm-Based Temperature Feature Extraction Method for Infrared Images of Electrical Equipment. Sensors.

[30] Cheng T., Gu J., Zhang X., Hua L., Zhao F. (2022). Multimodal image registration for power equipment using clifford algebraic geometric invariance. Energy Rep..

[31] Yan B., Zhao L., Miao K., Wang S., Li Q., Luo D. (2024). TGLFusion: A Temperature-Guided Lightweight Fusion Method for Infrared and Visible Images. Sensors.

[32] Faisal M.A.A., Mecheter I., Qiblawey Y., Fernandez J.H., Chowdhury M.E.H., Kiranyaz S. (2025). Deep learning in automated power line inspection: A review. Appl. Energy.

[33] Mendu B., Mbuli N. (2025). State-of-the-Art Review on the Application of Unmanned Aerial Vehicles (UAVs) in Power Line Inspections: Current Innovations, Trends, and Future Prospects. Drones.

[34] Rogalski A. (2003). Infrared detectors: Status and trends. Prog. Quantum Electron..

[35] Ray A. (2023). Radiation effects and hardening of electronic components and systems: An overview. Indian J. Phys..

[36] Li M. (2024). Systematic analysis of New Technologies and trends of infrared photodetectors. Highlights Sci. Eng. Technol..

[37] He R., Wang Y., Yan Z., Lu X. (2025). Infrared spectrum target recognition and positioning technology based on image segmentation algorithm. Discov. Artif. Intell..

[38] Feng W., Qin T., Tang X. (2024). Advances in Infrared Detectors for In-Memory Sensing and Computing. Photonics.

[39] Martyniuk P., Antoszewski J., Martyniuk M., Faraone L., Rogalski A. (2014). New concepts in infrared photodetector designs. Appl. Phys. Rev..

[40] An J., Wang B., Shu C., Wu W., Sun B., Zhang Z., Li D., Li S. (2020). Research development of 2D materials based photodetectors towards mid-infrared regime. Nano Sel..

[41] Long M., Wang P., Fang H., Hu W. (2018). Progress, challenges, and opportunities for 2D material based photodetectors. Adv. Funct. Mater..

[42] Zhou L., Huang Q., Xia Y. (2024). Plasmon-Induced Hot Electrons in Nanostructured Materials: Generation, Collection, and Application to Photochemistry. Chem. Rev..

[43] Rogalski A. (2012). History of infrared detectors. Opto-Electron. Rev..

[44] Mykkänen E., Lehtinen J.S., Grönberg L., Shchepetov A., Timofeev A.V., Gunnarsson D., Kemppinen A., Manninen A.J., Prunnila M. (2020). Thermionic junction devices utilizing phonon blocking. Sci. Adv..

[45] Balakrishnan G.K., Yaw C.T., Koh S.P., Abedin T., Raj A.A., Tiong S.K., Chen C.P. (2022). A Review of Infrared Thermography for Condition-Based Monitoring in Electrical Energy: Applications and Recommendations. Energies.

[46] Li T., Zhu C., Wang Y., Li M., Cao H., Li J., Yuan P., Bai H. (2026). FRNet: A lightweight feature reconfiguration network for infrared detection of substation equipment and instruments. Digit. Signal Process..

[47] Ding H., Zheng J. (2026). IDSE-YOLO: An Infrared Recognition Model for Substation Equipment. IEEE Access.

[48] Rogalski A. (2011). Recent progress in infrared detector technologies. Infrared Phys. Technol..

[49] Zhang P., Sun Y., Sun J., Wang S., Wang R., Zhang J. (2025). Progress in 2D Material-Based Infrared Photodetectors for Intelligent Vision Applications. Adv. Funct. Mater..

[50] Yan F., Wei Z., Wei X., Lv Q., Zhu W., Wang K. (2018). Toward High-Performance Photodetectors Based on 2D Materials: Strategy on Methods. Small Methods.

[51] Qing J., Wang S., Gu S., Lin L., Xie Q., Li D., Huang W., Guo J. (2024). Graphene-PbS Quantum Dot Heterostructure for Broadband Photodetector with Enhanced Sensitivity. Sensors.

[52] Mao R., Liu Z., Zhang Y., Ye J., Guo J. (2023). Ohmic-contacted WSe_2_/MoS_2_ heterostructures for broadband photodetector with fast response. Appl. Phys. Express.

[53] Chen J., Li Y., Cai J., Guo J. (2023). Tuning of graphene plasmons by ferroelectric superdomain for mid-infrared photodetector with high responsivity. Jpn. J. Appl. Phys..

[54] Guo J., Lin L., Li S., Chen J., Wang S., Wu W., Cai J., Liu Y., Ye J., Huang W. (2021). WSe_2_/MoS_2_ van der Waals heterostructures decorated with Au nanoparticles for broadband plasmonic photodetectors. ACS Appl. Nano Mater..

[55] Guo J., Li S., Chen J., Cai J., Gou X., Wang S., Ye J., Liu Y., Lin L. (2022). Tunable plasmonic devices by integrating graphene with ferroelectric nanocavity. Adv. Mater. Interfaces.

[56] Guo J., Gou X., Cai J., Wang S., Ye J., Chen J. (2022). Tunable mid-infrared absorber based on graphene/ferroelectric stacks with dual-band selectivity. Optik.

[57] Liu L., Liu Y., Gong T., Huang W., Guo J., Zhang X., Zhou S., Yu B. (2019). Graphene-based polarization-sensitive longwave infrared photodetector. Nanotechnology.

[58] Guo J., Liu Y., Lin Y., Tian Y., Zhang J., Gong T., Cheng T., Huang W., Zhang X. (2019). Simulation of tuning graphene plasmonic behaviors by ferroelectric domains for self-driven infrared photodetector applications. Nanoscale.

[59] Mirov S.B., Moskalev I.S., Vasilyev S., Smolski V., Fedorov V.V., Martyshkin D., Peppers J., Mirov M., Dergachev A., Gapontsev V. (2018). Frontiers of Mid-IR Lasers Based on Transition Metal Doped Chalcogenides. IEEE J. Sel. Top. Quantum Electron..

[60] Guo J., Li S., He Z., Li Y., Lei Z., Liu Y., Huang W., Gong T., Ai Q., Mao L. (2019). Near-infrared photodetector based on few-layer MoS_2_ with sensitivity enhanced by localized surface plasmon resonance. Appl. Surf. Sci..

[61] Han Q., Jiang Y., Liu X., Zhang C., Wang J. (2023). Visible to Mid-Infrared Photodetector Based on Black Phosphorous-MoS_2_ Van Der Waals Heterojunction. IEEE Photonics J..

[62] Li J., Xie Z., Zhao T., Li H., Wu D., Yu X. (2025). Metasurface-Enhanced Infrared Photodetection Using Layered van der Waals MoSe_2_. Nanomaterials.

[63] He X., Tan J., Hu T., Zhu L. (2025). Multimodal Segmentation for Photovoltaic Module Defect Detection. IEEE Access.

[64] Chen S., Li Z., Li S., Xu K., Ma N., Yue L., Jin X., Liu R., Dong Q., Li Q. (2025). Enhanced and Polarity-Switchable Photoresponse in MoS_2_ with Asymmetric Metal Contact via Pressure Band Engineering. Laser Photonics Rev..

[65] Enujekwu F.M., Zhang Y., Ezeh C.I., Zhao H., Xu M., Besley E., George M.W., Besley N.A., Do H., Wu T. (2021). N-doping enabled defect-engineering of MoS_2_ for enhanced and selective adsorption of CO_2_: A DFT approach. Appl. Surf. Sci..

[66] Kim J.W., Lee S.Y. (2024). Work function effect of metal electrodes on the performance of amorphous Si–Zn–Sn–O thin-film transistors investigated by transmission line method. J. Mater. Sci. Mater. Electron..

[67] Sun X., Abshire J.B., Krainak M.A., Lu W., Beck J.D., Sullivan W.W., Mitra P., Rawlings D.M., Fields R.A., Hinkley D.A. (2019). HgCdTe avalanche photodiode array detectors with single photon sensitivity and integrated detector cooler assemblies for space lidar applications. Opt. Eng..

[68] Akhavan N.D., Jolley G., Umana-Membreno G.A., Antoszewski J., Faraone L. (2015). Design of Band Engineered HgCdTe nBn Detectors for MWIR and LWIR Applications. IEEE Trans. Electron Devices.

[69] Chen H., Sun X., Lai K.W.C., Meyyappan M., Xi N. Infrared Detection Using an InSb Nanowire. Proceedings of the 2009 IEEE Nanotechnology Materials and Devices Conference.

[70] Cheah C.W., Tan L.S., Karunasiri R.P.G. Theoretical and experimental studies on the improvement of the response of n-type III-V QWIPs to TE mode infrared radiation. Proceedings of the COMMAD 2000 Proceedings, Conference on Optoelectronic and Microelectronic Materials and Devices.

[71] Kaynak C.B., Goeritz A., Durmaz E.C., Wietstruck M., Onat E., Ozcan A.S., Turkoglu E.R., Gurbuz Y., Kaynak M. Thermo-Mechanical Modeling and Experimental Validation of an Uncooled Microbolometer. Proceedings of the IEEE 20th RF System on Silicon in Wireless Era (RF SiW).

[72] Chen X. Black Phosphorus: Mid-Infrared Light-Emitting Properties and Devices. Proceedings of the 2022 20th International Conference on Optical Communications and Networks (ICOCN).

[73] Tankimanova A., Kang C.H., Alkhazragi O., Tang H., Kong M., Sinatra L., Lutfullin M., Li D., Ding S., Xu B. (2021). Colloidal PbS Quantum Dots for Visible-to-Near-Infrared Optical Internet of Things. IEEE Photonics J..

[74] Ma Z., Zhang S., Wang L., Rao Q. Global Context-Aware Method for Infrared Image Non-Uniformity Correction. Proceedings of the 2024 3rd International Conference on Artificial Intelligence and Computer Information Technology (AICIT).

[75] Qu W., Tang J. Multi-point Calibration Detecting Method of Non-linear Analogue Quantity. Proceedings of the 2009 Ninth International Conference on Hybrid Intelligent Systems.

[76] Goodall T., Bovik A.C., Vikalo H., Paulter N.G. Non-uniformity Correction of IR Images using Natural Scene Statistics. Proceedings of the 2015 IEEE Global Conference on Signal and Information Processing (GlobalSIP).

[77] Monir M.S., Maheshweri H.K. Random Noise Suppression in fMRI time-series Using Modified Spectral Subtraction. Proceedings of the 12th IEEE International Multitopic Conference.

[78] Cong S., Pu H., Wang X., Zhao Y. Application of Improved YOLOv5 in Infrared Image Recognition of Electrical Equipment. Proceedings of the 2023 8th Asia Conference on Power and Electrical Engineering (ACPEE).

[79] Zhou H.-X., Lai R., Liu S.-Q., Wang B.-J., Li Q. (2005). A new real-time processing system for the IRFPA imaging signal based on DSP&FPGA. Infrared Phys. Technol..

[80] Hudomalj U., Mandla C., Plattner M. FPGA Implementations for Real-Time Processing of High-Frame-Rate and High-Resolution Image Streams. Proceedings of the 2020 International Conference on Computing, Electronics & Communications Engineering (iCCECE).

[81] Torres-Huitzil C., Arias-Estrada M. (2004). Real-time image processing with a compact FPGA-based systolic architecture. Real.-Time Imaging.

[82] Wells C.C., Duncan E., Renshaw D. An FPGA based Prototyping Platform for Imager-On-Chip Applications. Proceedings of the 2004 IEEE International Conference on Field- Programmable Technology (IEEE Cat. No.04EX921).

[83] Fei Y., Jia X., Fan K.-C., Wang H., Liu X., Lu R. The design and implementation of a flexible FPGA/DSP based architecture for real-time image processing. Proceedings of the Fourth International Symposium on Precision Mechanical Measurements.

[84] Krishna D.B.H., Kumar C.A. A Novel Method Of Reconfigurable Image Processing Using Fpga. Proceedings of the International Conference on Electrical, Electronics, and Optimization Techniques (ICEEOT)—2016.

[85] Wu J.Q.M.J. An Investigation of FPGA Implementation for Image Processing. Proceedings of the 2010 International Conference on Communications, Circuits and Systems (ICCCAS).

[86] Annovi A., Berretta M., Crescioli F., Dell’Orso M., Giannetti P., Laurelli P., Maccarrone G., Sansoni A., Sartori L., Volpi G. A Fast FPGA-based Clustering Algorithm for Real Time Image Processing. Proceedings of the 2009 IEEE Nuclear Science Symposium Conference Record.

[87] Uzun I.S., Amira A., Bouridane A. (2005). FPGA implementations of fast Fourier transforms for real-time signal and image processing. IEE Proc. Vis. Image Signal Process..

[88] Yildirim M., Çinar A. Simultaneously Realization of Image Enhancement Techniques On Real-Time Fpga. Proceedings of the 2019 International Conference on Engineering and Technology (ICET).

[89] Zemcik P. Hardware Acceleration of Graphics and Imaging Algorithms Using FPGAs. Proceedings of the 2002 ACM/SIGDA 10th International Symposium on Field Programmable Gate Arrays.

[90] Cummins C., Petoumenos P., Wang Z., Leather H. End-to-End Deep Learning of Optimization Heuristics. Proceedings of the 2017 26th International Conference on Parallel Architectures and Compilation Techniques (PACT).

[91] Wang T., Xu Y.-Q. (2016). Photonic Structure-Integrated Two-Dimensional Material Optoelectronics. Electronics.

[92] Khan W.Z., Rehman M.H., Zangoti H.M., Afzal M.K., Armi N., Salah K. (2020). Industrial internet of things: Recent advances, enabling technologies and open challenges. Comput. Electr. Eng..

[93] Mait J.N., Euliss G.W., Athale R.A. (2018). Computational imaging. Adv. Opt. Photonics.

[94] Alabadi M., Habbal A., Wei X. (2022). Industrial Internet of Things: Requirements, Architecture, Challenges, and Future Research Directions. IEEE Access.

[95] Mirani T., Rajan D., Christensen M.P., Douglas S.C., Wood S.L. (2008). Computational imaging systems: Joint design and end-to-end optimality. Opt. Express.

[96] Malik P.K., Sharma R., Singh R., Gehlot A., Satapathy S.C., Alnumay W.S., Pelusi D., Ghosh U., Nayak J. (2021). Industrial Internet of Things and its Applications in Industry 4.0: State of The Art. Comput. Commun..

[97] Sisinni E., Saifullah A., Han S., Jennehag U., Gidlund M. (2018). Industrial Internet of Things: Challenges, Opportunities, and Directions. IEEE Trans. Ind. Inform..

[98] Kuzume K., Niijima K., Takano S. (2004). FPGA-based lifting wavelet processor for real-time signal detection. Signal Process..

[99] Kozlov A.V., Rodin V.G., Starikov R.S., Evtikhiev N.N., Cheremkhin P.A. (2023). Estimation of Camera’s Noise by Uniform Target Segmentation. IEEE Sens. J..

[100] Hamdoh A., Gao Y., Spires O., Brock N., Jiang L., Pau S. (2025). Short-wave infrared (SWIR) polarization imaging using division-of-focal-plane imaging polarimeter. Sci. Rep..

[101] Gundam M., Charalampidis D. Median Filter on FPGAs. Proceedings of the 44th IEEE Southeastern Symposium on System Theory.

[102] Hajabdollahi M., Samavi S., Karimi N. Error Compensation and Hardware Reduction of Fixed Point 2-D Gaussian Filter. Proceedings of the 9th Iranian Conference on Machine Vision and Image Processing.

[103] Luo J., Zhang Y. Infrared Image Enhancement Algorithm based on Weighted Guided Filtering. Proceedings of the 2021 IEEE 2nd International Conference on Information Technology, Big Data and Artificial Intelligence (ICIBA).

[104] Lan X., Shen H., Zhang L. An Adaptive Non-local Means Filter Based on Region Homogeneity. Proceedings of the 2013 Seventh International Conference on Image and Graphics.

[105] Andriyani A.D., Hawari Ghazali K., Riyadi S. Comparative Evaluation of CNN, LSTM, and Hybrid CNN-LSTM for Spatial-Spectral Classification of Hyperspectral Images. Proceedings of the 2025 International Conference on Information Technology and Computing (ICITCOM).

[106] Hao Z., Yu H., Yu W., Binbin L., Yi C., Cong W., Ning Z. Design and implementation of UAV inspection and control platform for transmission lines. Proceedings of the 2023 8th International Conference on Information Systems Engineering (ICISE).

[107] Chi Y., Li Y., Ye J., Li L., Xu K., Liang X. Study on Requirements for Test and Detection of Substation Inspection Robot. Proceedings of the 2022 5th International Conference on Energy, Electrical and Power Engineering (CEEPE).

[108] Omer Z.M., Fardoun A.A., Hussain A. Large Scale Photovoltaic Array Fault Diagnosis for Optimized Solar Cell Parameters Extracted by Heuristic Evolutionary Algorithm. Proceedings of the 2016 IEEE Power and Energy Society General Meeting (PESGM).

[109] Fernández A., Usamentiaga R., de Arquer P., Fernández M.Á., Fernández D., Carús J.L., Fernández M. (2020). Robust Detection, Classification and Localization of Defects in Large Photovoltaic Plants Based on Unmanned Aerial Vehicles and Infrared Thermography. Appl. Sci..

[110] Langåker H.-A., Kjerkreit H., Syversen C.L., Moore R.J.D., Holhjem Ø.H., Jensen I., Morrison A., Transeth A.A., Kvien O., Berg G. (2021). An autonomous drone-based system for inspection of electrical substations. Int. J. Adv. Robot. Syst..

[111] Gao H., Dekyi D., Metok M. (2025). Efficient unmanned aerial vehicle inspection and management of transmission lines in modern electric power enterprises. Energy Inform..

[112] Takaya K., Ohta H., Kroumov V., Shibayama K., Nakamura M. Development of UAV System for Autonomous Power Line Inspection. Proceedings of the International Conference on System Theory, Control and Computing (ICSTCC).

[113] Iversen N., Schofield O.B., Cousin L., Ayoub N., vom Bogel G., Ebeid E. Design, Integration and Implementation of an Intelligent and Self-recharging Drone System for Autonomous Power line Inspection. Proceedings of the 2021 IEEE/RSJ International Conference on Intelligent Robots and Systems (IROS).

[114] Nguyen V.N., Jenssen R., Roverso D. (2019). Intelligent Monitoring and Inspection of Power Line Components Powered by UAVs and Deep Learning. IEEE Power Energy Technol. Syst. J..

[115] Jalil B., Leone G.R., Martinelli M., Moroni D., Pascali M.A., Berton A. (2019). Fault Detection in Power Equipment via an Unmanned Aerial System Using Multi Modal Data. Sensors.

[116] Lu W., Li Q., Zhang W., Mei L., Cai D., Li Z. (2024). Management of power equipment inspection informationization through intelligent unmanned aerial vehicles. Artif. Life Robot..

[117] Dandurand P., Beaudry J., Hebert C., Mongenot P., Bourque J., Hovington S. All-weather autonomous inspection robot for electrical substations. Proceedings of the 2022 IEEE/SICE International Symposium on System Integration (SII).

[118] Lu S., Zhang Y., Su J. (2017). Mobile robot for power substation inspection: A survey. IEEE/CAA J. Autom. Sin..

[119] Zhang H., Su B., Meng H. (2017). Development and implementation of a robotic inspection system for power substations. Ind. Robot Int. J..

[120] Zou W., Shu X., Tang Q., Lu S. A Survey of the Application of Robots in Power System Operation and Maintenance Management. Proceedings of the 2019 Chinese Automation Congress (CAC).

[121] Bincai Z., Feifei W., Fucheng H. (2019). Technology and Application for Substation Automatic Inspection Based on “Robot +” Mode. J. Phys. Conf. Ser..

[122] Wang H., Li J., Zhou Y., Fu M., Yang S. Research on the Technology of Indoor and Outdoor Integration Robot Inspection in Substation. Proceedings of the 2019 IEEE 3rd Information Technology, Networking, Electronic and Automation Control Conference (ITNEC 2019).

[123] Zhu-mao L., Qing L., Tao J., Yong-xin L., Yu H., Yang B. Research on Thermal Fault Detection Technology of Power Equipment based on Infrared Image Analysis. Proceedings of the 2018 IEEE 3rd Advanced Information Technology, Electronic and Automation Control Conference (IAEAC 2018).

[124] Kim J.S., Choi K.N., Kang S.W. (2021). Infrared Thermal Image-Based Sustainable Fault Detection for Electrical Facilities. Sustainability.

[125] Zhu X., Li X., Yan F. (2018). Design and implementation for integrated UAV multi-spectral inspection system. 2018 Asia Conference on Energy and Environment Engineering (ACEEE 18), 19–21 January 2018, Shanghai, China.

[126] Yang L., Fan J., Liu Y., Li E., Peng J., Liang Z. (2020). A Review on State-of-the-Art Power Line Inspection Techniques. IEEE Trans. Instrum. Meas..

[127] Lin Y., Zhang W., Zhang H., Bai D., Li J., Xu R. An intelligent infrared image fault diagnosis for electrical equipment. Proceedings of the 2020 5th Asia Conference on Power and Electrical Engineering (ACPEE).

[128] Pu J., Zhang Q., Zhao W., Zhang W., Qin Z., Zhang Y. (2025). Research on refined UAV inspection method of wind/solar power stations based on YOLOv8. J. Eng. Appl. Sci..

[129] Rehman A., Park S.-J. (2020). State of the art two-dimensional materials-based photodetectors: Prospects, challenges and future outlook. J. Ind. Eng. Chem..

[130] Cui Q., Yang Y., Li J., Teng F., Wang X. (2017). Material and Device Architecture Engineering Toward High Performance Two-Dimensional (2D) Photodetectors. Crystals.

[131] Jiang J., Wen Y., Wang H., Yin L., Cheng R., Liu C., Feng L., He J. (2021). Recent Advances in 2D Materials for Photodetectors. Adv. Electron. Mater..

[132] Liu C., Guo J., Yu L., Li J., Zhang M., Li H., Shi Y., Dai D. (2021). Silicon/2D-material photodetectors: From near-infrared to mid-infrared. Light Sci. Appl..

[133] Gao M., Xu J., Tan A., Zu Z., Yang M., Wang J. (2017). Error correction based on micro-scanning preprocessing for an optical micro-scanning thermal microscope imaging system. Infrared Phys. Technol..

[134] Zhao Q., Yuan W., Qu J., Cheng Z., Peng G.-D., Yu C. (2022). Optical Fiber-Integrated Metasurfaces: An Emerging Platform for Multiple Optical Applications. Nanomaterials.

[135] Yang Y., Seong J., Choi M., Park J., Kim G., Kim H., Jeong J., Jung C., Kim J., Jeon G. (2023). Integrated metasurfaces for re-envisioning a near-future disruptive optical platform. Light Sci. Appl..

[136] Dhanabalan S.C., Ponraj J.S., Zhang H., Bao Q. (2016). Present perspectives of broadband photodetectors based on nanobelts, nanoribbons, nanosheets and the emerging 2D materials. Nanoscale.

[137] Wu P., Wang Z., Fan Z., He T., Zhu H., Wang Y., Li Q., Fu X., Wang F., Wang P. (2021). Next-generation machine vision systems incorpo. InfoMat.

[138] Yang C., Wu Y., Zhong C., Wang X., Li L., Guo J., Huang W., Liu Y. (2025). Compact and voltage-tunable surface plasmon polariton-based optical neural networks. Opt. Lett..

[139] Pouyanfar S., Sadiq S., Yan Y., Tian H., Tao Y., Reyes M.P., Shyu M.-L., Chen S.-C., Iyengar S.S. (2018). A Survey on Deep Learning. ACM Comput. Surv..

[140] Li N., Ma L., Yu G., Xue B., Zhang M., Jin Y. (2023). Survey on Evolutionary Deep Learning: Principles, Algorithms, Applications, and Open Issues. ACM Comput. Surv..

[141] Verhelst M., Moons B. (2017). Embedded Deep Neural Network Processing. IEEE Solid-State Circuits Mag..

[142] Rani M., Dhok S.B., Deshmukh R.B. (2018). A Systematic Review of Compressive Sensing: Concepts, Implementations and Applications. IEEE Access.

[143] Li L., Fang Y., Liu L., Peng H., Kurths J., Yang Y. (2020). Overview of Compressed Sensing: Sensing Model, Reconstruction Algorithm, and Its Applications. Appl. Sci..

[144] Donoho D.L. (2006). Compressed sensing. IEEE Trans. Inf. Theory.

[145] Zhao F., Zhang C., Geng B. (2024). Deep Multimodal Data Fusion. ACM Comput. Surv..

[146] Peng Y., Zhai Z., Feng M. (2024). RGB-D Salient Object Detection Based on Cross-Modal and Cross-Level Feature Fusion. IEEE Access.

[147] Peng Y., Qi J., Huang X., Yuan Y. (2017). CCL: Cross-modal Correlation Learning with Multi-grained Fusion by Hierarchical Network. IEEE Trans. Multimed..

[148] Ekren N., Karagöz Z., Şahin M. (2023). A Review of Line Suspended Inspection Robots for Power Transmission Lines. J. Electr. Eng. Technol..

[149] Li L., Li N., Li Y., Zhang B., Zhao J., Zhang C., Dai Z. A State-of-the-Art Survey of the Robotics Applied for the Power Industry in China. Proceedings of the 2016 4th International Conference on Applied Robotics for the Power Industry (CARPI).

[150] Zhang T., Dai J. (2020). Electric Power Intelligent Inspection Robot: A Review. J. Phys. Conf. Ser..

[151] Varghese B., Wang N., Barbhuiya S., Kilpatrick P., Nikolopoulos D.S. Challenges and Opportunities in Edge Computing. Proceedings of the 2016 IEEE International Conference on Smart Cloud (SmartCloud).

[152] Cao K., Liu Y., Meng G., Sun Q. (2020). An Overview on Edge Computing Research. IEEE Access.

[153] Yao J., Zhang S., Yao Y., Wang F., Ma J., Zhang J., Chu Y., Ji L., Jia K., Shen T. (2022). Edge-Cloud Polarization and Collaboration: A Comprehensive Survey for AI. IEEE Trans. Knowl. Data Eng..

[154] Bao G., Guo P. (2022). Federated learning in cloud-edge collaborative architecture: Key technologies, applications and challenges. J. Cloud Comput..

[155] Hu L., Sun G., Ren Y. (2020). CoEdge: Exploiting the Edge-Cloud Collaboration for Faster Deep Learning. IEEE Access.

[156] Esmaeili N., Vafamehr M., Rezaei H., Khaki A. (2023). Identifying the Influencing Factors of Design Standardization in the Industrialization of Architecture: Review and Future Directions. Creat. City Des..

[157] Blind K., von Laer M. (2021). Paving the path: Drivers of standardization participation at ISO. J. Technol. Transf..

[158] Huneeus F., Rogerson R. (2023). Heterogeneous Paths of Industrialization. Rev. Econ. Stud..

